# Magnetic Ionotropic Hydrogels Based on Carboxymethyl Cellulose for Aqueous Pollution Mitigation

**DOI:** 10.3390/gels9050358

**Published:** 2023-04-24

**Authors:** Andra-Cristina Enache, Ionela Grecu, Petrisor Samoila, Corneliu Cojocaru, Valeria Harabagiu

**Affiliations:** Laboratory of Inorganic Polymers, “Petru Poni” Institute of Macromolecular Chemistry, 41A Grigore Ghica Voda Alley, 700487 Iasi, Romania

**Keywords:** carboxymethyl cellulose, manganese ferrite, sodium dodecyl sulfate, ionotropic hydrogels, magnetic beads, biosorbent, dye adsorption, molecular docking

## Abstract

In this work, stabilized ionotropic hydrogels were designed using sodium carboxymethyl cellulose (CMC) and assessed as inexpensive sorbents for hazardous chemicals (e.g., Methylene Blue, MB) from contaminated wastewaters. In order to increase the adsorption capacity of the hydrogelated matrix and facilitate its magnetic separation from aqueous solutions, sodium dodecyl sulfate (SDS) and manganese ferrite (MnFe_2_O_4_) were introduced into the polymer framework. The morphological, structural, elemental, and magnetic properties of the adsorbents (in the form of beads) were assessed using scanning electron microscopy (SEM), energy-dispersive X-ray analysis, Fourier-transform infrared spectroscopy (FTIR), and a vibrating-sample magnetometer (VSM). The magnetic beads with the best adsorption performance were subjected to kinetic and isotherm studies. The PFO model best describes the adsorption kinetics. A homogeneous monolayer adsorption system was predicted by the Langmuir isotherm model, registering a maximum adsorption capacity of 234 mg/g at 300 K. The calculated thermodynamic parameter values indicated that the investigated adsorption processes were both spontaneous (Δ*G* < 0) and exothermic (Δ*H* < 0). The used sorbent can be recovered after immersion in acetone (93% desorption efficiency) and re-used for MB adsorption. In addition, the molecular docking simulations disclosed aspects of the mechanism of intermolecular interaction between CMC and MB by detailing the contributions of the van der Waals (physical) and Coulomb (electrostatic) forces.

## 1. Introduction

Water is a scarce and indispensable resource for our lives [1]. Notwithstanding this, the reality of global climate change and human activities have altered the quality of drinking water in recent decades [2,3]. Domestic, agricultural, and industrial activities, as well as hospital sewage and power generation, are just a few of the major contributors to water pollution [4]. Therefore, various hazardous compounds (organic, inorganic, radioactive pollutants, pathogens, nutrients, and others) are directly discharged into the water bodies [5,6]. As a consequence, more than 50 types of diseases arise as a result of drinking water contamination worldwide, which frequently leads to high mortality rates, particularly among children [7].

The synthetic dye-based industries (e.g., textiles, paper, pharmaceuticals, leather, and plastic materials) are one of the most concerning sectors when it comes to water contamination [8]. Every year, approximately 1,000,000 tons of different organic dyes are produced, and their release into the environment can endanger all life forms [8,9]. Among these, the cationic dyes have a tendency to be more harmful than the anionic dyes, as they have high tinctorial values (1 mg/L) [10] and can decompose into carcinogenic amine compounds under anaerobic conditions [11]. Methylene blue (MB) is a well-known heterocyclic cationic dye widely used in the pharmaceutical and medical fields, as well as in the textile and plastic industries [9,12]. However, MB is extremely destructive and a major threat to human health upon ingestion (damaging the neurological and visual systems) [13]. In addition, exposure to high doses of MB (>2 mg/kg) can cause other associated conditions, such as cardiopulmonary affections, nausea, Heinz body formation, tissue necrosis, jaundice, quadriplegia, breathing difficulties, and gastritis infections [9,14]. Additionally, MB’s non-biodegradability and accumulation capacity make it highly problematic for the environment and aquatic life [12,15].

In order to reduce wastewater MB levels, different remediation methods have been investigated so far: adsorption [8], membrane separation [16], photodegradation [12], electrochemical treatment [17], coagulation-flocculation [18], constructed wetlands [19] and others. Besides being efficient, low-cost, and easy to design and operate, adsorption is one of the most attainable techniques in cationic dye removal. Other advantages of this method are the high selectivity for dyes of different concentrations and the relatively simple regeneration processes [20].

Therefore, in terms of economic feasibility and environmental impact, researchers have proven in recent years the efficiency of polysaccharide-based adsorbents (such as chitosan [8], alginate [21], kappa-carrageenan [22], cellulose [23], and others) for MB removal from aqueous solutions. Among these, carboxymethyl cellulose (CMC) was found to be a promising derivative of cellulose (the most prevalent natural polysaccharide in nature), as it is an anionic and water-soluble polyelectrolyte, ideal for cationic dye adsorption [23,24]. The main benefits of using CMC-based adsorbents for MB removal are their biodegradability, eco-friendliness, cost-effectiveness, gel-forming properties, and negatively charged surfaces (–CH_2_COOH groups), which are suitable for binding cationic dyes through electrostatic interactions [25,26,27]. However, CMC has been shown to have poor mechanical qualities and limited adsorption capacity when used alone in wastewater applications. In consequence, physical and/or chemical modifications of the CMC or the incorporation of different types of particles in the CMC matrix (e.g., graphene oxide, metals, clays) in order to obtain composite materials have demonstrated improved capacities for MB adsorption [25,26,28].

Relying on the previously stated interest, this work proposes the development of sustainable, cost-effective, and non-toxic CMC-based sorbent in the form of beads with high added value through: (i) the preparation of hydrodynamically stabilized ionotropic hydrogels by cross-linking with iron cations; (ii) using sodium dodecyl sulfate (SDS) as a foaming agent and the freeze-drying method to improve the adsorption capacity; iii) facilitating the sorbent recovery through easy magnetic separation by adding manganese ferrite (MnFe_2_O_4_) to the polymer framework. As far as we are aware, this study is the first to describe the preparation of an ionotropic hydrogel based on Fe^3+^ cross-linked carboxymethyl cellulose with magnetic properties (provided by MnFe_2_O_4_ particles) in the form of freeze-dried beads. Furthermore, this is the first report on the use of these materials in the adsorption of cationic dyes, such as methylene blue, highlighting the adsorption mechanism, intermolecular interactions between MB and CMC, and sorbent-sorbate interaction energies through molecular docking computations.

## 2. Results and Discussion

### 2.1. Spinel Ferrite and Magnetic Beads Preparation

As a result of their low energy consumption, simple preparation procedure, and use of inexpensive and readily available raw materials, physical hydrogel formulations are considered promising materials for environmental applications [29]. However, environmental applications require certain aspects, such as the use of natural, biocompatible, and non-toxic materials, avoiding chemical leakage, increasing pollutant adsorption capacities, facilitating the recovery, reuse, etc.

**Figure 1:**
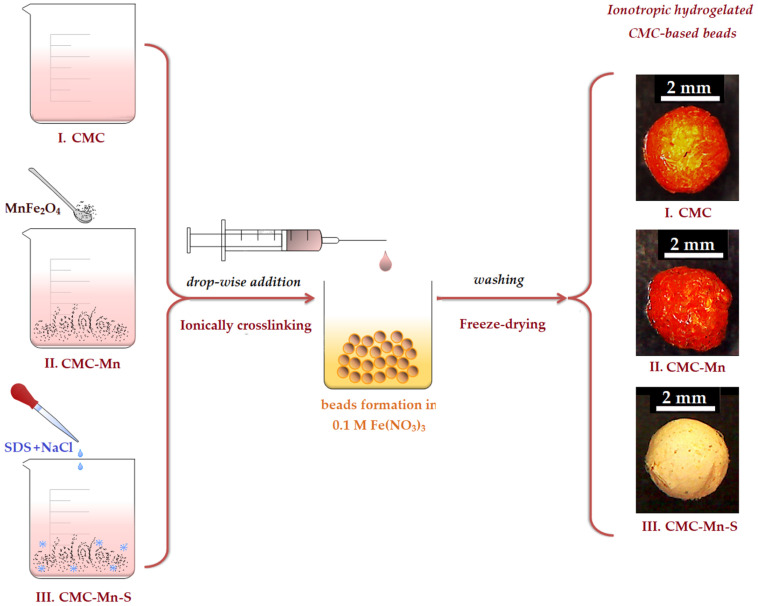
Schematic representation CMC-based bead preparation, where CMC is the 3% carboxymethyl cellulose solution (I); CMC-Mn is the magnetic dispersion of manganese spinel ferrite (MnFe_2_O_4_) in the CMC solution (II); and CMC-Mn-S represents the mixture after the addition of sodium dodecyl sulfate (SDS) into the magnetic dispersion (III). The beads obtained have the same annotation as the initial solutions.

Due to its eco-friendly properties and the abundance of reactive functional groups –COO^−^, carboxymethyl cellulose (CMC) has been used in this work as a polymeric matrix for the preparation of adsorbent beads (as described in Table 1). A possible source of toxicity in ionotropic hydrogels is the leakage of crosslinking ions, which occurs especially when crosslinking with divalent ions (e.g., Ca^2+^, Mg^2+^) [30]. To avoid this drawback and to confer superior stability to the CMC-based hydrogels, trivalent Fe^3+^ cations were used to bind the carboxylic groups. The beads were prepared in an easy and inexpensive manner, as graphically represented in Figure 1, by drop-wise addition of CMC-based mixtures in a solution containing Fe^3+^ cations (0.1 M Fe(NO_3_)_3_). According to the literature [29], the instantaneous cross-linking mechanism involves the attachment of each Fe^3+^ trivalent cation to three polymeric chains of CMC, substituting Na^+^ ions. Thus, the feasibility of selecting the ionotropic gelation of carboxymethyl cellulose (CMC) with iron cations (predominately stabilized by electrostatic interactions) can be considered a key factor for large-scale usage at minimal application cost.

One of the problems encountered in wastewater treatment generally deals with sorbent recovery from aqueous media [31]. As compared to traditional separation technologies (settling, centrifugation, and membrane filtration [32]), the ease of magnetic separation was investigated in this study by the introduction of manganese spinel ferrite (MnFe_2_O_4_) in the polymeric matrix. The MnFe_2_O_4_ inorganic particles were selected due to their extraordinary magnetic properties [33] and were synthesized by a flexible method, such as the sol-gel auto-combustion technique. This type of process uses an exothermic, self-sustaining anionic redox reaction in an aqueous solution of metal salts. The advantage of using nitrate salts as precursors is that they provide a water-soluble, low temperature oxidant source for synthesis. These salts (in this study, Mn^2+^ and Fe^3+^ nitrates) are classified as oxidizers. Citric acid serves as both a reductant and an oxygen source for the self-combustion stage.

In addition to the presence of –COO^−^ functional groups in the CMC matrix that aid in the binding of cationic pollutants (e.g., Methylene Blue), the hydrogel matrix’s ability to swallow dye solutions should also allow dye molecules to adsorb on its surface via van der Waals interactions or hydrogen bonds [34]. Moreover, to improve the adsorption capacity of the CMC hydrogel matrix, attempts were made to adjust the chemical structure and pore formation by adding different ratios of sodium dodecyl sulfate (SDS) surfactant and NaCl salt (see chemical composition in Table 1). The use of NaCl with SDS (Figure S1), before the crosslinking process, aimed to form larger micellar aggregates (the salt bridges stabilizing the micelles and increasing their size) [29]. The removal of these aggregates by washing with ethanol should lead to the appearance of a porous structure, with pore connections. Finally, the magnetic beads prepared by modification of CMC with SDS (CMC-Mn-S1, CMC-Mn-S2, and CMC-Mn-S3, respectively) were characterized by comparison with non-magnetic (CMC) and/or magnetic unmodified beads (CMC-Mn). After evaluation of adsorption capacities, the most promising material was subjected to in-depth examination, in order to provide a sustainable mitigation measure for methylene blue removal from aqueous solutions.

### 2.2. Manganese Ferrite Characterization

**Figure 2:**
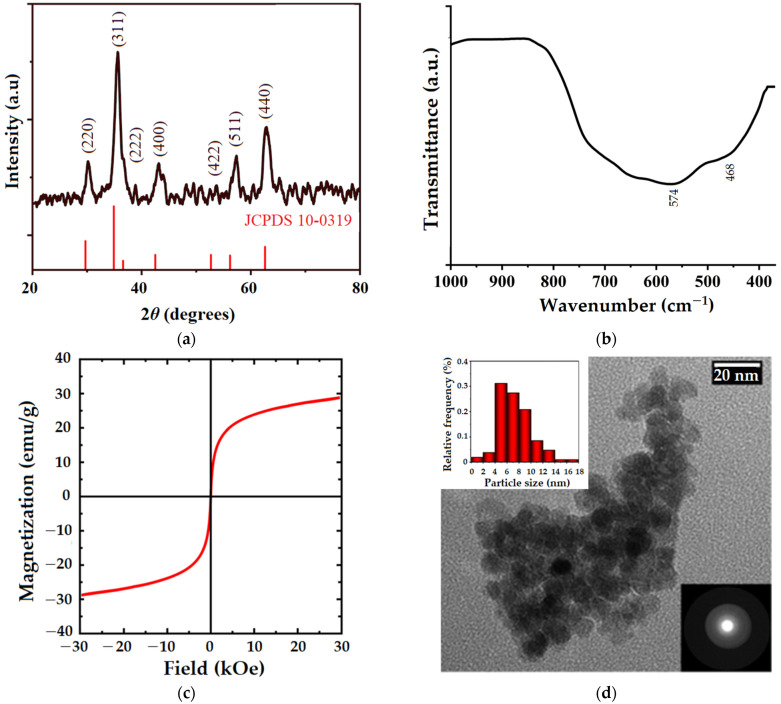
Manganese spinel ferrite analysis by (**a**) X-ray diffraction pattern compared to the standard XRD pattern of MnFe_2_O_4_ (JCPDS card no. 10-0319); (**b**) FT-IR spectrum; (**c**) VSM analysis; (**d**) representative TEM image, particle size distribution (upper left corner), and selected electron diffraction pattern (lower right corner).

X-ray diffraction (XRD), Fourier-transform infrared spectroscopy (FTIR), vibrating-sample magnetometer (VSM), and transmission electron microscopy (TEM) were used to characterize the manganese ferrite (MnFe_2_O_4_) obtained by the sol-gel auto-combustion technique, as shown in Figure 2. According to the XRD pattern in Figure 2a, the MnFe_2_O_4_ sample is characterized by a crystalline structure. Peaks observed at 30.25°, 35.64°, 36.90°, 43.02°, 53.7°, 57.2°, and 62.85° confirm the pure cubic spinel structure of manganese ferrite (according to JCPDS card no. 10-0319) and correspond to the (220), (311), (222), (400), (422), (511), and (440) diffraction planes, respectively [35,36]. Figure 2b shows the FTIR spectra of MnFe_2_O_4_ registered in the range of 1000–400 cm^−1^, which is in close agreement with the XRD findings about the spinel structure of the analyzed sample. Therefore, the band observed at 574 cm^−1^ is attributed to the vibration of the metal-oxigen bond in the tetrahedral sites of the spinel matrix, and the band at 468 cm^−1^ corresponds to the vibration of the metal-oxigen bond in the octahedral positions [35]. VSM was used to evaluate the magnetic properties of manganese ferrite nanoparticles at room temperature (Figure 2c). Considering the fact that the magnetization loop has no hysteresis, the obtained ferrite can be classified as superparamagnetic, with a magnetization value of 28.77 emu/g. This is also supported by coercivity and remanence values that are almost zero, indicating that the magnetization practically disappears with the removal of the external magnetic field. Similar behavior was also observed when manganese ferrite was synthesized by the co-precipitation technique [35]. The morphological features of MnFe_2_O_4_ nanoparticles were assessed by TEM, as highlighted in Figure 2d. Thus, the almost spherical-shaped nanoparticles can be observed, with a high tendency to agglomerate due to their magnetic properties. Based on the TEM image, particle size distribution was determined using Image J software (version 1.45s, National Institute of Health, Bethesda, MI, USA). According to the inset histogram (Figure 2d, upper left corner), the MnFe_2_O_4_ nanoparticles were found to be smaller than 18 nm in size. The selected electron diffraction pattern (Figure 2d, lower right corner) shows individualized, blurry rings, suggesting once again the crystallinity of the sample, as it is also shown in the XRD pattern.

### 2.3. CMC-Based Beads Characterization

#### 2.3.1. Morphological Analysis

**Figure 3:**
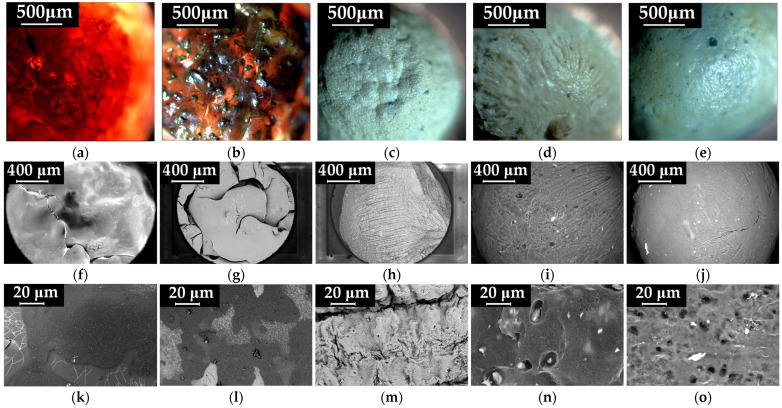
Surface morphology images of CMC (**a**,**f**,**k**), CMC-Mn (**b**,**g**,**l**), CMC-Mn-S1 (**c**,**h**,**m**), CMC-Mn-S2 (**d**,**i**,**n**), and CMC-Mn-S3 (**e**,**j**,**o**) beads, obtained by PoLM (**a**–**e**) and SEM (**f**–**o**) microscopy, respectively.

The surface and cross-section morphology of the CMC-based beads were analyzed by polarized light microscopy (PoLM) and scanning electron microscopy (SEM), as depicted in Figure 3 and Figure S2 (in Supplementary Materials), respectively. The PoLM images reveal the main modifications that occur in the CMC beads surface morphology (Figure 3a) induced by the addition of inorganic MnFe_2_O_4_ (Figure 3b) and SDS surfactant (Figure 3c–e). Thus, Figure 3a shows the uncompressed morphology of an unmodified CMC hydrogelated matrix, which allows the nanometric cellulosic fibrils to be seen. Moreover, the CMC beads are characterized by an orange color, provided by the cross-linking of the carboxylic groups with Fe^3+^ cations (also visible in Figure 1). These features are consistent with the cross-sectional morphology of the CMC granules (Figure S2, in Supplementary Materials), which show large pores and thin walls, typical of cellulosic hydrogels obtained by the freeze-drying process [37].

In comparison to the pristine CMC beads, the addition of ferrite nanoparticles into the polysaccharide matrix induced an irregular surface morphology of the CMC-Mn beads with a proclivity for the agglomeration of the magnetic component (Figure 3b). However, the cross-section of the beads revealed no significant morphological changes, as shown in Supplementary Materials (Figure S2b). When SDS and NaCl are added during the gelation process, the obtained CMC-Mn-S1, CMC-Mn-S2, and CMC-Mn-S3 beads acquire a whitish color and a specific morphology, both at the surface (Figure 3c–e) and in cross-section (Figure S2c–e). In addition, for SDS-modified beads, better dispersion of the magnetic nanoparticles in the polymer matrix was observed.

For an in-depth analysis of the morphological modifications of the beads, SEM microscopy was used (Figure 3f–j). It can be noticed that the images obtained by SEM are consistent with their analogues provided by polarized light microscopy (Figure 3a–e). Despite the cross-sectional morphology obtained by the freeze-drying process (large, elongated pores), the CMC and CMC-Mn beads present a non-porous and brittle surface morphology as a result of cross-linking with iron cations (Figure 3f,g,k,l).

Other authors demonstrated that the anionic surfactant SDS self-assembles into micelles in aqueous environments and forms micellar aggregates, mainly when NaCl salt is added in excess [29]. As expected, its use in the CMC hydrogel matrix led to the appearance of a porous surface, which should provide increased efficiency and adsorption capabilities to the obtained beads. Furthermore, the use of SDS led to a compact cross-sectional porous morphology (Figure S2h–j), which was needed for improved mechanical properties of the beads. This can be explained by the fact that the presence of SDS large micelles led to an in-depth, interconnected, crosslinked network. Note that the presence of iron is well observed in the bead’s cross-section, as shown in Figure S2m–o. By comparison with CMC-Mn-S1, which has a folded surface, increasing the SDS content led to spherical beads with a highly porous and smoother surface, as observed for CMC-Mn-S2 and CMC-Mn-S3 samples.

The CMC polymeric matrix modifications were confirmed by changes occurring at the level of constituent elements, as highlighted in Supplementary Materials (Figure S3). The elemental mapping allowed the visualization of the elements in the analyzed samples. Figure S3d (corresponding to CMC beads) reveals the presence of carboxymethyl cellulose elements (C and O) as well as the crosslinking agent (Fe). It can be noticed that the Fe element is uniformly distributed as a result of the cross-linking process (Figure S3g). By comparison with pristine CMC beads, the insertion of manganese spinel ferrite was confirmed by the presence of the Mn element in both CMC-Mn and CMC-Mn-SDS samples (Figure S3e,h and f,I, respectively). Moreover, the presence of S, Na, and Cl elements confirms the addition of SDS and NaCl salts during the CMC gelling process (Figure S3f).

#### 2.3.2. Structural Modifications

**Figure 4:**
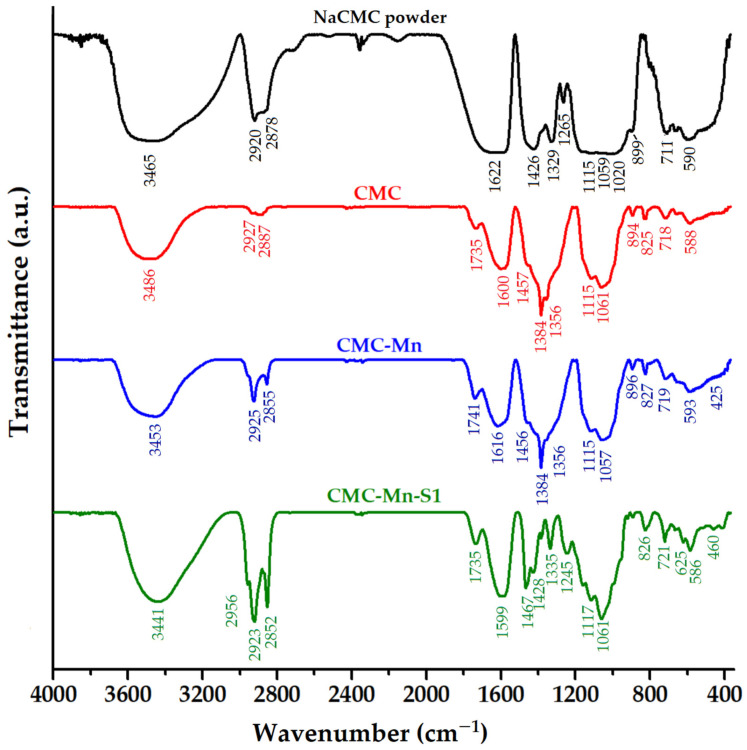
FTIR spectra of carboxymethyl cellulose powder (raw CMC powder) and of the investigated beads (CMC, CMC-Mn, and CMC-Mn-S1).

The structural properties of the CMC-based hydrogelated beads and the interactions at the polysaccharide functional groups were investigated using FTIR analysis, as shown in Figure 4. The spectrum of sodium carboxymethyl cellulose powder (NaCMC) revealed characteristic absorption bands: the broad band at 3465 cm^−1^ is related to O–H stretching vibrations (alcohol and intermolecular bonding); bands at 2920 cm^−1^ and 2878 cm^−1^ correspond to C–H (methylene) symmetric and asymmetric stretching vibrations, respectively; the typical bands for asymmetric and symmetric stretching of the –COO^−^Na^+^ carboxylate are found at 1622 cm^−1^ and 1426 cm^−1^, respectively; the band at 1329 cm^−1^ is attributed to C–H bending, coupled with—OH bending, while the 1265 cm^−1^ band is given by C–H deformation; the region between 1200 and 930 cm^−1^ overlaps the stretching vibrations of the anhydroglucose units (C–O–C) with the C–O vibration from primary (C6–OH at 1020 cm^−1^) and secondary alcohols (C2–OH at 1115 cm^−1^ and C3–OH at 1059 cm^−1^); the glycosidic bonds (β1–4) vibration appears at 899 cm^−1^; the band at 711 cm^−1^ is due to the monosubstituted out of plane =C–H bending, and the band at 590 cm^−1^ corresponds to C–C–O and O–C–O in plane deformation vibrations [23,37,38,39,40].

By comparison with pristine NaCMC, the cross-linking process with iron cations induced visible structural modifications in CMC beads (Figure 4). The adsorption bands corresponding to O–H (3486 cm^−1^) and C–H stretching vibrations (2927 and 2887 cm^−1^) were shifted to higher wavenumbers. The appearance of a new peak in the CMC spectrum (1735 cm^−1^) is due to the carbonyl (–C=O) stretch, from the protonated –COOH group). This confirms the interactions between carboxylic groups in CMC and iron cations. Similar observations were made when CMC was crosslinked with Al^3+^ ions [29] or when a sodium carboxymethyl cellulose/sodium alginate blend was crosslinked with Fe^3+^ ions [41]. Shifting the absorption bands of –COO^−^ asymmetric and symmetric stretching to lower wavenumbers (1600 and 1384 cm^−1^_,_ respectively), with the disappearance of the 1426 cm^−1^ band (assigned to carboxyl groups as salts), also suggests the existence of chemical interactions between carboxylate groups and iron cations [42]. The appearance of the shoulder at 1457 cm^−1^ is due to –CCH and –OCH bending vibrations of the pyranose ring [43]. The shifting of ΔO–H band from 1329 cm^−1^ to 1356 cm^−1^ in the CMC beads could also indicate the participation of polysaccharide hydroxyl groups in the formation of chelating structures with Fe^3+^. This trend is also supported by the disappearance of the C6–OH band (1020 cm^−1^), which suggests the interaction of the primary hydroxyl groups, and the increase in intensity of the C3–OH band (1061 cm^−1^). The region 900–500 cm^−1^ has also undergone a few changes following the cross-linking of the CMC polysaccharide.

The CMC-Mn spectrum resembles the CMC spectrum (Figure 4), with slight displacement of the absorption bands. However, the presence of manganese spinel ferrite is confirmed by the presence of Fe–O and Mn–O characteristic absorption bands, which can be observed in the range of 600–400 cm^−1^. By comparison with the CMC and CMC-Mn spectra, the use of SDS surfactant led to specific modifications. The bands corresponding to CMC-Mn structure are easily shifting, but new bands appear due to surfactant chemical structure, such as 2956 cm^−1^ (from CH_3_ asymmetric stretching vibrations), 1467 and 1061 cm^−1^ (asymmetric and symmetric stretching νO=S=O of sulfonate groups), and 1245 cm^−1^ due to C–H bending vibrations [29]. However, the increase in intensity in the regions 3400–3000 and 1740–1500 could indicate a more reactive surface (–OH and –COO^−^ groups).

#### 2.3.3. Magnetic Properties

**Figure 5:**
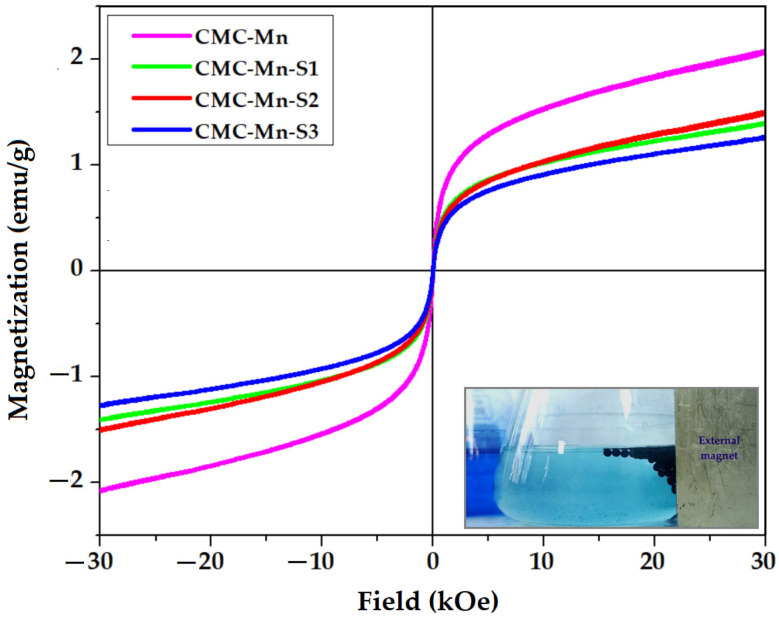
VSM analysis of manganese ferrite-loaded CMC beads (the inset image shows the separation of the MB-loaded beads from the aqueous solution under an external magnetic field).

The magnetization curves of MnFe_2_O_4_-loaded CMC beads (CMC-Mn and CMC-Mn-S1-3) were determined by using VSM, as shown in Figure 5. All the CMC-based magnetic beads presented magnetization loops similar to those of pure manganese ferrite (Figure 2c). Thus, the investigated beads keep their superparamagnetic character, as indicated by the lack of hysteresis as well as zero coercivity and remanence. However, the decrease of the magnetization values (registered at 30 kOe) from 28.77 emu/g (pure MnFe_2_O_4_) to values ranging from 1.2 to 2.1 emu/g should be mentioned, as given in Table 1. This can be attributed to the use of small amounts of ferrite (only 10%) in the polysaccharide matrix. For the same reason, the addition of SDS led to an additional proportional decrease in magnetization. However, an exception is made by the CMC-Mn-S2 beads, which have a slightly higher magnetization when compared to the beads with less SDS (CMC-Mn-S1), although the initial trend was the opposite. The magnetization values and the superparamagnetic properties of the beads are sufficient for providing easy separation from a solution when an external field is applied, even when the beads are charged with the adsorbed dye (as the lower right corner image from Figure 5 demonstrates).

### 2.4. Adsorption Tests of Methylene Blue (MB) Cationic Dye

#### 2.4.1. Batch Adsorption Screening Test

**Figure 6:**
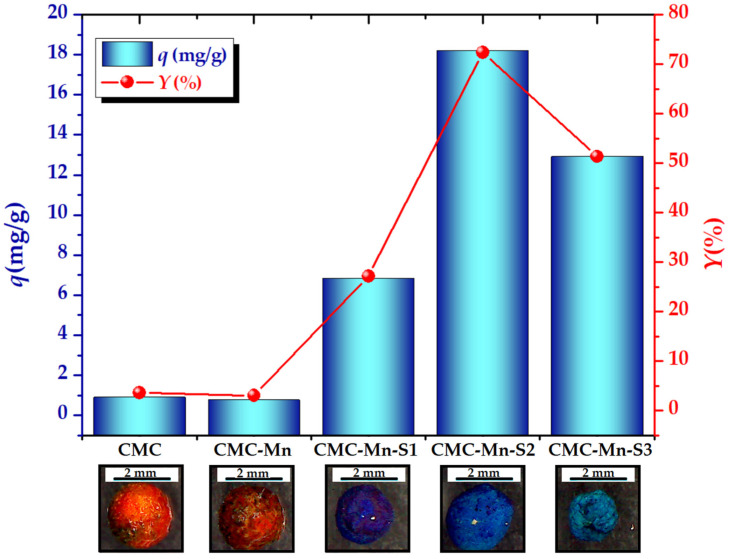
Batch screening test (adsorption capacity—*q*, mg/g, and color removal efficiency—*Y*, %) for Methylene Blue (MB) removal from aqueous solutions at room temperature (sorbent dose of 2 g/L, contact time of 1 h, MB initial concentration of 50 mg/L); macroscopic images of dried CMC-based beads after the adsorption process.

In order to determine the adsorption performance of MB cationic dye from aqueous solutions under the same conditions onto the carboxymethyl cellulose-based beads (CMC, CMC-Mn, and CMC-Mn-S1-3), a batch adsorption screening test [44] was carried out. The preliminary results regarding the adsorption capacities (*q*, mg/g) and dye removal efficiency (*Y*, %) are highlighted in Figure 6. As expected, the addition of SDS surfactant during the gelling process of CMC led to increased adsorption capacities as compared with CMC and CMC-Mn beads (Table 1). This is attributed to the electrostatic repulsions of the -O-SO_3_^−^ groups from the SDS surfactant molecules and polyanionic CMC chains in the gelation process, which favor the formation of a more porous structure [29]. After SDS removal, the electrostatic interactions between the carboxylic groups of CMC (present on the beads’ surface and within their pores) and MB molecules are expected to improve. Of these, CMC-Mn-S2 magnetic beads displayed the best adsorption performance, with the highest values of adsorption capacity (18.22 ± 0.23 mg/g) and color removal efficiency (72.38 ± 0.92%). The beads were dried after the adsorption assay, and their macroscopic images are shown in Figure 6. It should be noted that the color is more intense in CMC-Mn-S1 due to the shrinkage of the beads, whereas CMC-Mn-S2 retains its shape even after drying. The adsorption properties of CMC-Mn-S2 beads can also be correlated with their surface morphology (Figure 3d,i,n). In addition to these, the higher value of magnetization among SDS-containing samples recommends the use of CMC-Mn-S2 beads as adsorbents for subsequent tests (adsorption kinetics and isotherms).

#### 2.4.2. Kinetics and Isotherms

**Figure 7:**
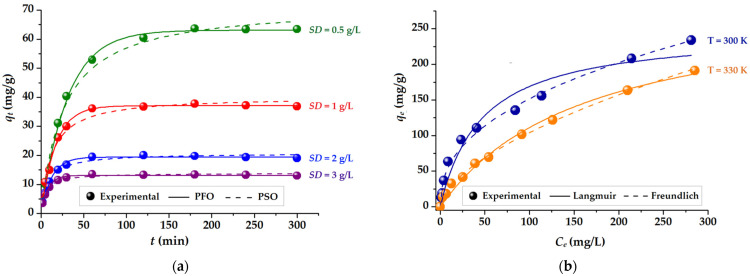
Adsorption of Methylene Blue onto CMC-Mn-S2 adsorbent: (**a**) experimental data for different sorbent doses (0.5–3 g/L) and mathematical kinetic models (pseudo-first order and pseudo-second order); (**b**) experimental data at two temperatures (300 and 330 K) and isotherm models (Langmuir and Freundlich).

The effect of the contact time on the removal of MB from aqueous solutions is assessed since the dye’s rate of adsorption is an important factor in the accurate evaluation of the adsorbent. As depicted in Figure 7a, the adsorption capacity (*q*, mg/g) of CMC-Mn-S2 hydrogelated beads was evaluated over time for different sorbent doses (*SD* = 0.5–3 mg/L). As expected, the adsorption of MB cationic dye is increasing with contact time, regardless of the sorbent dosage employed, due to the enhanced driving forces. As a result, a three-stage adsorption tendency was noticed [45], which is most noticeable when smaller amounts of sorbent are used. In the initial phase, a rapid increase is observed in the first 30 min, mainly due to the fast adsorption of MB on the CMC-Mn-S2 beads surface. Then, in the second stage (30–180 min), a slower adsorption is observed, which is more apparent when employing a lower dose of sorbent. The active adsorption sites on the surface of the beads are often limited at this point, and the MB molecules in the solid phase start to exhibit repulsive forces [29]. Following this stage, the dye molecules seek to diffuse into the pores of the beads and are slowly adsorbed by the deeper active sites until equilibrium is reached. In the third stage (>180 min), the adsorption capacity reaches a steady state due to the saturation of the carboxyl groups with MB molecules. This leads to the attainment of adsorption equilibrium.

The impact of the sorbent dose (*SD*) on the adsorption capacity (*q*, mg/g) is also depicted in Figure 7a. Hence, it can be shown that after 6 h of the experiment, the increase in the sorbent dose led to a decrease in the adsorption capacity of MB. Consequently, a sorbent dose of 0.5 g/L resulted in the highest adsorption capacity of 63.46 mg/g, which was then decreased to 36.88 mg/g for *SD* = 1 g/L, 19.04 mg/g for *SD* = 2 g/L, and finally to 12.99 mg/g for *SD* = 3 g/L. This trend was also found by others [46] and is mainly attributed to the presence of excessive active sites as the sorbent dose is incremented.

For an in-depth investigation of the adsorption rate of the adsorption process [29], the experimental data were analyzed using two kinetic models: Lagergren’s pseudo-first-order model (PFO) and Ho’s pseudo-second-order model (PSO) [47]. Predictions according to the two models are represented in Figure 7 (by solid and dashed lines), while the corresponding non-linear equations and kinetic parameters are detailed in Table S1. The goodness-of-fit was employed to determine the better agreement of predicted data with the experimental ones by calculating the chi-squared (*χ^2^)* statistical test (smaller values are better), as described by Equation (S1) in Supplementary Materials. According to Figure 7a and *χ^2^* values from Table S1, the PFO model best describes the adsorption kinetics of MB onto CMC-Mn-S2 hydrogelated beads [47]. Moreover, this model’s assessment of the theoretical equilibrium adsorption capacity (q_e_^(calc)^) is consistent with the experimental findings (q_e_^(obs)^). A better fit given by PFO (compared to PSO) might be attributed to the fact that the separation process most probably involves a single main step, controlled by adsorption rather than diffusion. Similar findings were reported by other authors that investigated the adsorption of Cu(II) ions onto semi-interpenetrated polymer networks [48] or when the MB adsorption was evaluated on walnut-shell-based cellulosic materials [49].

The adsorption isotherms were investigated at 300 K and 330 K, respectively, in order to evaluate the relationship between the equilibrium concentrations (*C_e_*, mg/L) of the cationic dye MB and the CMC-Mn-S2 adsorption capacities at equilibrium (*q_e_,* mg/g). Considering the results obtained in the kinetic study (Figure 7a) for the determination of the adsorption isotherms, a contact time of 300 min was used to ensure the achievement of the adsorption equilibrium. The experimental data given in Figure 7b indicates a decrease in adsorption capacity at equilibrium with increasing temperature, from 234 mg/g (at 300 K) to 191 mg/g (at 330 K). This can be explained by the fact that when the temperature rises, the MB molecules acquire sufficient kinetic energy to overcome the electrostatic attraction and detach from the sorbent surface [45]. The faster and increased adsorption of MB on the CMC-Mn-S2 beads at lower temperatures can be an advantage when it comes to environmental applications (temperatures that are more similar to those of the environment).

The adsorption process and the affinity of CMC-Mn-S2 beads toward MB molecules were investigated by using the Langmuir and Freundlich isotherm models [29,50]. The experimental data were interpolated, and the calculated predictions are shown in Figure 7 with solid and dashed lines. Additionally, the chi-square (χ^2^) test was performed, and the corresponding values are given in Table S2 (Supplementary Materials), along with the equations and parameters of the isotherms. Thus, the data in Table S2 show that the Langmuir equation is the most appropriate model for the investigated systems, suggesting monolayer dye adsorption (a smaller value of χ^2^). The Langmuir isotherm model predicted a homogeneous monolayer adsorption system, indicating a finite number of equivalent sites and no interactions between MB molecules at the surface of the CMC-Mn-S2 beads [29]. Furthermore, a higher value of the Langmuir constant (*K_L_* = 0.021 L/mg) indicates stronger interactions and increased affinity between the surface of the CMC-Mn-S2 beads and cationic dye molecules at a lower temperature (300 K). A further analysis can be expressed in terms of the dimensionless equilibrium parameter (*R_L_*), to reveal the favorable adsorption of the Langmuir isotherm, in conformity with Equation (1):

(1) $$R_{L}=\frac{1}{1+K_{L}C_{0}},$$

where *K_L_* represents the Langmuir constant and *C*_0_ is the initial dye concentration. The *R_L_* is usually determined in order to confirm the nature of the adsorption process: irreversible adsorption (*R_L_* = 0); favorable adsorption (0 < *R_L_* < 1) and linear unfavorable adsorption (*R_L_* = 1) [50,51]. As shown in Table S2, the calculated values of the *R_L_* factor were 0.373 (300 K) and 0.614 (330 K) for initial dye concentrations (*C*_0_) ranging from 10 to 400 mg/L. These results confirmed the favorable adsorption of MB cation dyes onto hydrogelated CMC-Mn-S2 adsorbent.

Furthermore, the Dubinin-Radushkevich (D-R) isotherm model was used to determine the type of adsorption process by calculating the mean free energy of adsorption, E_S_ (kJ/mol), as given in Table S2. The values of E_S_ make it possible to understand the nature of the adsorption process (physical, ion exchange, or chemisorption). Hence, values between 8 and 16 kJ/mol reflect an ion exchange process, while E < 8 kJ/mol characterizes a physical adsorption mechanism [52]. According to D-R data given in Table S2, the mean free energies of the investigated systems were 10.91 kJ/mol (at 300 K) and kJ/mol (at 330 K). This suggests that the adsorption of MB dye onto the CMC-Mn-S2 surface occurs mainly via strong electrostatic interactions (ion exchange mechanisms between the negatively charged carboxylic (–COO^−^) groups of the adsorbent and positively charged amino groups from MB).

**Figure 8:**
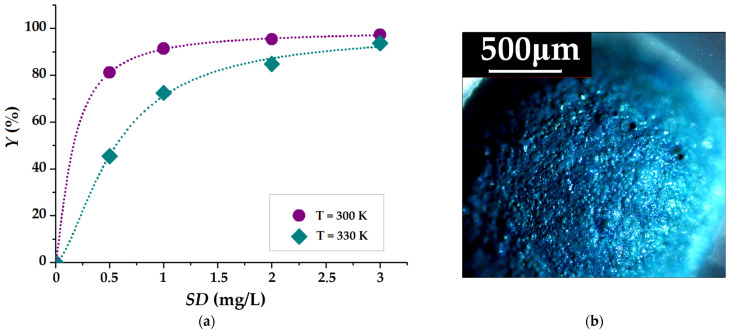
(**a**) Removal efficiency at equilibrium (%) of MB from aqueous solutions (*C*_0_ = 50 mg/L) as a function of sorbent dose (*SD*); (**b**) Image obtained by polarized light microscopy of the CMC-Mn-S2 surface after MB adsorption.

Figure 8a highlights the influence of sorbent dose (*SD*) and temperature on MB removal efficiency (*Y*). As discussed in the kinetic assay (Figure 7a), the increase in *SD* led to a decrease in adsorption capacity (*q*, mg/g). However, Figure 8a shows that by increasing the *SD* (at both 300 and 330 K temperatures), the removal efficiency of the MB dye from the aqueous solution is increasing. This behavior is explained by the fact that increasing the *SD* for a fixed number of MB molecules (50 mg/L) implies the availability of more adsorption sites. As a consequence, a larger amount of MB is adsorbed from the aqueous solution, but the amount of MB per unit mass of CMC-Mn-S2 adsorbent decreases. Because the MB dye is a well-known exogenous fluorophore [53], its homogeneous adsorption on the CMC-Mn-S2 surface was highlighted by using polarized light microscopy (Figure 8b). Moreover, the penetration into the depth of the beads of MB molecules was proved by the cross-section image (given in Figure S4, Supplementary Materials) due to the porous network created by the SDS surfactant’s introduction during the gelation process. These results are in agreement with SEM morphology (discussed in Section 2.3.1). In addition, the presence of iron is well observed in the MB-loaded beads cross-section (Figure S4), confirming the absence of iron leakage during the adsorption process and the increased stability of the polymeric hydrogelated matrix.

Furthermore, we performed a comparative literature analysis on the efficacy of our CMC-based beads in comparison to existing CMC-based composite materials used to remove MB from aqueous solutions (maximum adsorption capacities are given in Table S3, Supplementary Materials). In contrast to CMC-based composites in the form of membranes [54], films [55], or (nano)particles [26,56], biosorbents in the form of aerogels [27,57] or (micro)granules [25,29,58,59] demonstrated higher MB adsorption capacities (between 75 and 245 mg/g). However, the maximum experimental MB adsorption capacity obtained in this study (234 mg/g) suggests that CMC-Mn-S2 beads are suitable materials for efficient removal of MB from aqueous solutions.

More than that, to demonstrate the suitability of CMC-Mn-S2 beads for cationic dye adsorption, other organic dyes were tested. Crystal violet (CV) and brilliant green (BG), two additional cationic dyes frequently employed in pharmaceutical applications [49,60], were examined for their ability to bind to these CMC-Mn-S2. Thus, Figure S5 points out the ability of CMC-Mn-S2 to adsorb high amounts of CV (62.4 mg/g) and BG (51.9 mg/g) from aqueous solutions after a contact time of 60 min. It should be noted that all adsorption studies were carried out at neutral pH ≈ 6 (by dissolving MB powder in distilled water), first to be suited for environmental applications and secondly because the adsorption capacities at more acidic or alkaline pH were found to be lower, as shown in Figure S6.

#### 2.4.3. Thermodynamics

A detailed understanding of the underlying energy changes associated with the adsorption process can be obtained by determining thermodynamic parameters such as Gibbs free energy (Δ*G*), enthalpy (Δ*H*), and entropy (Δ*S*). The values of these thermodynamic parameters were calculated following the approach reported in our previous study [49]. For the adsorption process reported in this work, the thermodynamic parameters are summarized in Table 2.

Taking into account the negative values of the Gibbs free energy (Δ*G* < 0), it can be stated that the adsorption processes of MB onto an adsorbent have a spontaneous (exergonic) character. Because a more negative value of Δ*G* (−17,671 kJ/mol) was recorded at 300 k compared to the value at 330 K (−15,981 kJ/mol), it can be mentioned that a better affinity between the CMC-Mn-S2 adsorbent and MB molecules occurs at lower temperatures. It is important to note that these results are in agreement with the previous adsorption isotherm study (Figure 7b). Table 2 shows the negative enthalpy value (Δ*H* < 0), suggesting exothermic effects of the adsorption. Additionally, the negative values of entropy indicate a rearrangement of the adsorption of MB on the CMC-Mn-S2 surface. As a result of the association between organic dye molecules and adsorbents, it appears that randomness at the solid-liquid interface decreases. A similar trend was observed when MB was adsorbed onto a CMC-based composite membrane [54].

#### 2.4.4. Desorption Assay and Re-Use Test

The desorption experiments (in ethanol and acetone), followed by the MB re-adsorption (*q*, mg/g) on CMC-Mn-S2 recovered beads are discussed in detail in Supplementary Materials (Figure S7). However, it should be mentioned that desorption efficiency in acetone reaches 93%, implying a subsequent MB re-adsorption capacity of 48.6 mg/g (close to the initial adsorption values of about 52.9 mg/g). The possibility of being re-used is beneficial in environmental applications, and CMC-Mn-S2 can be considered an efficient adsorbent for removing MB from wastewater.

### 2.5. Molecular Docking

The molecular docking computations were performed to detail the intermolecular interactions between the cationic dye and the CMC-Mn-S2 adsorbent. The MB dye molecule (in cationic form) was used as the ligand for molecular docking, and the CMC oligomer (a tetramer) was used as the receptor. The YASARA-Structure program was used to create the structures of both the ligand (MB) and the receptor (CMC tetramer). The structures were then subjected to geometry optimization at the molecular mechanics theory level using the YASARA force field.

Then, for the molecular docking simulations, the optimal conformations (3-D structures) of the receptor and ligand were used. In order to achieve this, 100 docking poses were tested at the YASARA force field level. During molecular docking simulations, the receptor was regarded as a rigid body, whereas the ligand (the MB dye molecule) was treated as a flexible body. The YASARA-Structure program’s “AutoSMILES” method was used to automatically generate the parameters for the modeled structures.

**Figure 9:**
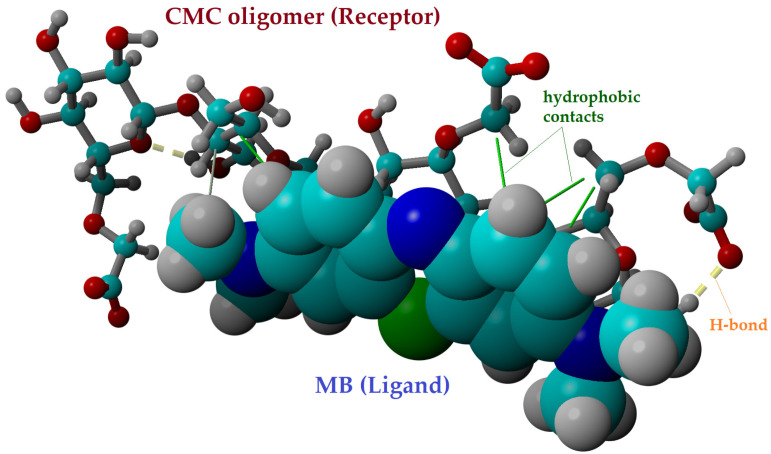
Molecular docking outcomes showing the best pose of the docked complex between the CMC tetramer receptor and MB ligand, as well as their interaction mode (*E_b_* = −3.92 kcal/mol and *K_d_* = 1.33 mM).

Figure 9 displays the outcomes of the molecular docking in the optimal docked complex pose, highlighting the intramolecular interactions between the ligand (MB) and the receptor (CMC tetramer). The hydrogen bonds (H-bonds), which are represented as dotted yellow lines in Figure 9, were found to be present inside the CMC receptor. Moreover, the intermolecular interactions between the MB molecule and the CMC tetramer are based on hydrophobic interactions (represented as solid green lines). These hydrophobic interactions can be linked to the physical adsorption of MB dye onto CMC (based on van der Waals forces). The docked complex CMC/MB presented a binding energy (*E_b_*) of −3.92 kcal/mol and a dissociation constant (*K_d_*) of 1.33 mM, according to computational calculations. The dissociation of the docked complex is feasible due to the considerable value of the dissociation constant (*K_d_* = 1.33 mM), which indicates that the connection between the MB ligand and the CMC receptor is not very strong.

In order to take into account the contributions of the van der Waals and Coulomb (electrostatic) forces, the interaction energies between the ligand (MB dye) and CMC receptor were also computed at the level of the YASARA force field (molecular mechanics theory). According to the theoretical results, electrostatic Coulomb forces (Δ*E_CL_* = −33.25 kcal/mol) are dominant compared to van der Waals forces (Δ*E_vdW_* = −21.92 kcal/mol) in the mechanism of intermolecular interaction between MB dye and CMC oligomer. The docking results for the CMC/MB system were in good agreement with the D-R isotherm results, which suggested a mechanism of adsorption based on ion exchange (i.e., electrostatic interactions).

## 3. Conclusions

By ionically cross-linking with iron cations, sodium carboxymethyl cellulose (CMC) was used to create stabilized ionotropic hydrogels in the form of beads. In order to facilitate the magnetic separation of sorbents from aqueous solutions, MnFe_2_O_4_ nanoparticles (synthesized by the sol-gel auto-combustion method) were introduced into the hydrogelated matrix. In addition, SDS surfactant was used as a pore generator (porogen) to increase the adsorption capacity and hydrodynamic stability of the CMC-based beads. Therefore, magnetic beads were prepared by modification of CMC with SDS surfactant (CMC-Mn-S1, CMC-Mn-S2, and CMC-Mn-S3, respectively), and characterized (morphologically, structurally, and magnetically) by comparison with non-magnetic (CMC) and magnetic unmodified beads (CMC-Mn). After evaluation of adsorption capacities, CMC-Mn-S2 was found to be the most promising material and was further subjected to in-depth examination. The adsorption kinetics were best described by the PFO model, and the maximum adsorption capacity was registered at lower temperatures (234 mg/g at 300 K). The isotherm data were best interpolated by the Langmuir isotherm model, suggesting a homogeneous monolayer adsorption mechanism. According to the thermodynamic parameters, it was found that the investigated adsorption processes occurred spontaneously (Δ*G* < 0), revealing an exothermic nature (Δ*H* < 0). After immersion in acetone, the sorbent was recovered (93% desorption efficiency) and successfully re-used for another adsorption test. In addition, molecular docking suggested that the interaction between MB dye and CMC was mainly based on electrostatic Coulomb forces (Δ*E_CL_* = −33.25 kcal/mol). These appeared between the negatively charged carboxylic (–COO^−^) groups of the adsorbent and the positively charged amino groups in the MB molecule. Due to the fact that CMC-based physical hydrogel formulations fulfill certain aspects, such as the use of natural, biocompatible, and non-toxic materials, reduced chemicals leakage, increased pollutant adsorption capacities, and ease of recovery, the investigated hydrogelated beads can be considered a promising sorbent for environmental applications.

## 4. Materials and Methods

### 4.1. Materials

Manganese nitrate (Mn(NO_3_)_2_ ∙ 6H_2_O), iron nitrate (Fe(NO_3_)_3_ ∙ 9H_2_O), and citric acid (C_6_H_8_O_7_ ∙ H_2_O) were purchased from Merck Chemical (Saint Louis, MO, USA) and employed in manganese spinel ferrite synthesis. Sodium carboxymethyl cellulose (sodium salt, average M.W. 90000, DS = 0.7) was acquired from Acros Organics (Geel, Belgium), while sodium dodecyl sulfate (SDS), sodium chloride (NaCl), and Methylene Blue (MB) were supplied by Merck Chemical (Saint Louis, MO, USA). Ethanol was provided by Chemical Company (Iasi, Romania). These analytically graded materials were used without further purification.

### 4.2. Manganese Spinel Ferrite and CMC-Based Magnetic Beads Preparation

#### 4.2.1. Synthesis of Manganese Spinel Ferrite (MnFe_2_O_4_)

The manganese spinel nanostructures were prepared by the sol-gel auto-combustion method [32]. In our case, the metal nitrate solutions (Mn(NO_3_)_2_ and Fe(NO_3_)_3_) were blended in stoichiometric proportions. Then the chelating agent was added. Thus, the molar ratio of cations to citric acid was 1:3. The procedure continued with the stirring and heating of the resulting solution in a water bath at 80 °C. Further, the sol developed into a dark-brown, porous, dry gel (Figure S8a). By gradually elevating the temperature up to 350 °C, self-propagating combustion was observed. This process was complete when loose black powder was formed (Figure S8b).

#### 4.2.2. CMC-Based Beads Preparation

The preparation of CMC-based beads took place in an easy manner, as graphically represented in Figure 1. In a first phase, a stock solution of carboxymethyl cellulose (CMC) was prepared by dissolving 3% sodium carboxymethyl cellulose (NaCMC) in distilled water under continuous stirring at 45 °C for 24 h. In a second phase, the required amount of manganese spinel ferrite (10% by reference to CMC mass) was dispersed in 2 mL of distilled water by ultrasonication for 20 min (Emmi 12 HC, 100% ultrasonic efficiency). The obtained dispersion was added to the CMC solution, and the mixture was kept for another 60 min in the ultrasonic bath till homogenization was attained (CMC-Mn). In the third stage, in order to obtain porous magnetic beads, three different ratios of sodium dodecyl sulfate (SDS) and NaCl were added to the CMC-Mn mixture (CMC/SDS of 3/0.2, 3/0.4, and 3/0.8), while the amount of NaCl was maintained constant (4% by reference to polymer mass). The three mixtures containing SDS were named with increasing SDS concentrations: CMC-Mn-S1, CMC-Mn-S2, and CMC-Mn-S3, respectively.

The mixtures obtained in each phase were further subjected to an ionotropic gelling process by drop-wise addition in a 0.1 M Fe(NO_3_)_3_ bath using a LEGATO^®^ 100 Syringe Pump (KD Scientific, Holliston, MA, USA). To remove the excess surfactant, the hydrogelated beads were carefully cleaned with ethanol multiple times before being washed with distilled water. In a final step, each type of bead was subjected to a freeze-drying process for 24 h (Christ Alpha 3–4 LSCbasic, Osterode, Germany), finally obtaining CMC, CMC-Mn, CMC-Mn-S1, CMC-Mn-S2, and CMC-Mn-S3 beads.

### 4.3. Characterization Methods

#### 4.3.1. MnFe_2_O_4_ Characterization

The XRD pattern of the synthetized manganese ferrite was registered using a diffractometer Bruker D8 ADVANCE (Bruker, Karlsruhe, Germany) in the 2θ domain (20–80°), with a scanning step of 0.02° and a recording rate of 1 °/min. Infrared spectroscopy was performed in the wavenumber range of 4000–400 cm^−1^, using a Bruker Vertex 70 FTIR spectrometer (Ettlingen, Germany) and the KBr pellet technique (at room temperature). Magnetic measurements were made on a LakeShore 8607 vibrating sample magnetometer (VSM, Shore Cryotronics, Westerville, OH, USA) at ambient temperature. The samples were demagnetized in an alternating field prior to each test. A Hitachi High-Tech HT7700 transmission electron microscope (TEM) (Hitachi High Technologies Company, Tokyo, Japan) was used to examine the morphology and microstructure. After being dispersed in acetone, the samples underwent 30 min of ultrasonication, drop casting on copper grids covered with Ted Pella carbon (Redding, CA, USA), and vacuum-assisted 60 °C drying.

#### 4.3.2. CMC-Based Beads Characterization

The surface and cross-section morphology of CMC-based beads were investigated using a scanning electron microscope (SEM) with a resolution of 4 nm at 30 kV (FEI Quanta 200, Brno, Czech Republic). The chemical composition of the membranes was determined using the Quanta 200 system’s energy-dispersive X-ray spectrometer (EDX). Using a polarized optical microscope (PoLM, Leica Microsystems, Wetzlar, Germany), beads morphology and MB dye adsorption onto the biosorbents were additionally observed. Structural and magnetic properties were investigated by FTIR and VSM (see Section 4.3.1).

#### 4.3.3. Adsorption and Dessorption Assays of Methylene Blue Cationic Dye

The adsorption of Methylene Blue (MB) onto CMC-based beads was investigated using an orbital shaker-incubator, Biosan ES-20/60 (Riga, Latvia). A double-beam UV-VIS spectrophotometer, Hitachi U-U-2910 (Hitachi High Technologies Company, Tokyo, Japan), was used to measure the concentration of the cationic dye in the aqueous solutions at a wavelength of 664 nm.

The batch adsorption screening test was performed by immersing 0.1 g of adsorbent (*SD* = 2 g/L) in 50 mL of MB solution (*C*_0_ = 50 mg/L) for 1 h. The final concentration was measured, and the adsorption capacity (*q_t_*, mg/g) and removal efficiency (*Y_t_*, %) were calculated according to Equations (2) and (3):

(2) $$q\;\left(\mathrm{mg}/g\right)=\frac{\left(C_{0}-C_{t}\right)V}{m\cdot 1000},$$

(3) $$Y_{t}\;\left(\%\right)=\frac{C_{0}-C_{t}}{C_{0}}\cdot 100,$$

where *q_t_* (mg/g) is the amount of dye that has been adsorbed at time *t*, *C*_0_ and *C_t_* (mg/L) denote the dye concentrations in the initial and final solutions (after contact time *t*), respectively; *V* (mL) refers to the volume of the immersion medium, and *m* (g) represents the weight of the CMC-based beads. It should be noted that for the batch adsorption screening test, three measurements were performed for each system, and the values of adsorption capacity and removal efficiency shown in Figure 6 represent their average. The magnetic beads with the best adsorption performance were further employed in kinetics and isotherm studies. The kinetic adsorption was realized at T = 300 K, by immersion of different sorbent doses (corresponding to 0.5, 1, 2, and 3 g/L) in 50 mL of MB solution placed in the orbital shaker (initial concentration of 50 mg/L, 140 rpm, 6 h). The final dye concentration in solution was determined by taking aliquots from the dye solutions at various predetermined contact times, and the adsorption capacity at time *t* (*q_t_*, mg/g) was calculated (based on Equation (2)). The adsorption isotherms were measured at 300 K and 330 K using the same sorbent dose (0.5 g/L) and varying the initial dye solution concentrations (10 and 400 mg/L). The contact time was set at 6 h to ensure the adsorption process’s equilibrium at 180 rpm. The adsorption capacity (*q_e_*, mg/g) and removal efficiency (*Y*, %) of MB dye at equilibrium from aqueous solution were calculated according to Equations (2) and (3) (by replacing the *C_t_* concentration at time *t* with the *C_e_* concentration at equilibrium).

After the adsorption processes, 0.025 g of used CMC-Mn-S2 were dried and subjected to desorption tests (0.025 g of sorbent each in 50 mL of ethanol, respectively in 50 mL of acetone), being left overnight (at a temperature of 300 K and 150 rpm). The desorption efficiency (%) was calculated as the ratio between the amount of MB desorbed and the amount of MB initially adsorbed (multiplied by 100). After recovering the samples (Figure 7b), they were subjected to a MB re-adsorption cycle, according to Equation (2).

#### 4.3.4. Molecular Modeling

The molecular modeling simulations were performed on a Dell Precision workstation T7910 with 32 CPU threads. In this regard, the molecular docking computations were performed by using the AutoDock VINA algorithm [61] inbuilt into the YASARA-Structure program package (v.20.8.23) for modeling and visualization [62,63].

## Figures and Tables

**Table 1:** Chemical composition and characteristics of CMC-based beads.

Beads Code	Beads Composition ^1^				Beads Size ^2^ (mm)	Magnetization at 30 kOe (emu/g)	Adsorption Capacity (*q*, mg/g)	Removal Efficiency (*Y*, %)
	CMC (g)	MnFe_2_O_4_ (% *w*/*w*)	SDS (% *w*/*w*)	NaCl (% *w*/*w*)				
CMC	3	−	−	−	2.60 ± 0.28	−	0.9	3.60
CMC-Mn	3	10	−	−	2.23 ± 0.36	2.07	0.77	3.06
CMC-Mn-S1	3	10	0.2	4	1.76 ± 0.25	1.39	6.82	27.10
CMC-Mn-S2	3	10	0.4	4	2.09 ± 0.24	1.50	18.22	72.38
CMC-Mn-S3	3	10	0.8	4	1.97 ± 0.27	1.26	12.92	51.33

^1^ all the beads were prepared by using a stock solution of 3% CMC, and the crosslinking bath was 0.1 M Fe(NO_3_)_3_. ^2^ average value (from 25 measurements) ± standard deviation.

**Table 2:** Values of various thermodynamic parameters for the adsorption of the MB cationic dye onto the CMC-Mn-S2 adsorbent.

Temperature	Δ*G* (kJ/mol)	Δ*H* (kJ/mol)	Δ*S* (J/K.mol)
300 K	−17.671	−34.572	−56.337
330 K	−15.981		−56.337

## Data Availability

Not applicable.

## Supplementary Materials

The following supporting information can be downloaded at: https://www.mdpi.com/article/10.3390/gels9050358/s1, Figure S1: Chemical structure and 3D Conformer of SDS molecule; Figure S2: Cross-section morphology of CMC (a–c); CMC-Mn (d–f); CMC-Mn-S1 (g–i); CMC-Mn-S2 (j–l); CMC-Mn-S3 (m–o), obtained by polarized light microscopy and SEM, respectively; Figure S3: SEM images of CMC (a), CMC-Mn (b) and CMC-Mn-S1 (c) on the beads surface; corresponding elemental mapping and weight percentage of the constituent elements of the analyzed SEM surfaces (d–f), and the distribution of Fe (g), and Mn element in samples (h–i); Figure S4: Image obtained by polarized light microscopy of CMC-Mn-S2 cross-section after MB adsorption; Figure S5: Adsorption capacities of Methylene Blue (MB), Crystal Violet (CV), and Brilliant Green (BG) onto CMC-Mn-S2 beads (experimental conditions: *SD* = 0.025, *C*_0_ = 50 mg/g, *V* = 50 mL, *t* = 60 min); Figure S6: Adsorption capacities of Methylene Blue (MB) from aqueous solutions of pH 2 (adjusted with 0.1 M H_2_SO_4_) and pH 11 (adjusted with 0.1 M NaOH); experimental conditions: *SD* = 0.025, *C*_0_ = 50 mg/g, *V* = 50 mL, *t* = 60 min; Figure S7: (a) Desorption efficiency of MB in ethanol and acetone (experimental conditions: *SD* = 0.025, *V* = 50 mL, *t* = 24 h); and re-adsorption capacities (*q*, mg/g) of MB onto the recovered adsorbent (experimental conditions: *SD* = 0.025, *C*_0_ = 50 mg/g, *V* = 50 mL, *t* = 60 min); (b) macroscopic images of CMC-based beads after desorption in ethanol (left) and acetone (right). Figure S8: Representative images of the obtained (a) xerogel and (b) loose dark nanoparticles of manganese ferrite; Table S1: Kinetic models non-linear equations and parameters for MB dye adsorption onto CMC-Mn-S2 adsorbent, using different sorbent doses (*SD*); experimental conditions: T = 300 K, *C*_0_ = 50 mg/L; Table S2: Isotherm models, equations and parameters for MB dye adsorption onto CMC-Mn-S2 adsorbent (contact time: *t* = 300 min); Table S3. Comparison of the maximum adsorption capacities (q_e_^(obs)^) of CMC-based composite materials for retention of MB cationic dye.
