# Supplementary material for: Magnetic Ionotropic Hydrogels Based on Carboxymethyl Cellulose for Aqueous Pollution Mitigation
Source: Gels. 2023 Apr 24;9(5):358. doi: 10.3390/gels9050358 (PMC10217587; doi:10.3390/gels9050358)
Supplement: Supplementary file 1 [file gels-09-00358-s001.zip › gels-2348742-supplementary.docx]

**Supplementary Materials**

Magnetic Ionotropic Hydrogels Based on Carboxymethyl Cellulose for Aqueous Pollution Mitigation

Andra-Cristina Enache, Ionela Grecu, Petrisor Samoila*, Corneliu Cojocaru and Valeria Harabagiu

Laboratory of Inorganic Polymers, “Petru Poni” Institute of Macromolecular Chemistry, 41A Grigore Ghica Voda Alley, 700487 Iasi, Romania

***** Correspondence: samoila.petrisor@icmpp.ro


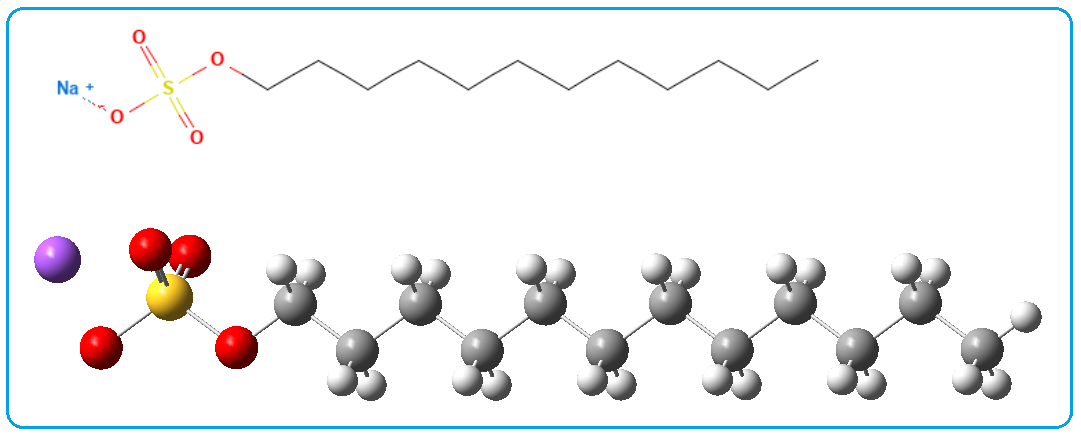


**Figure S1.** Chemical structure and 3D Conformer of SDS molecule.

| 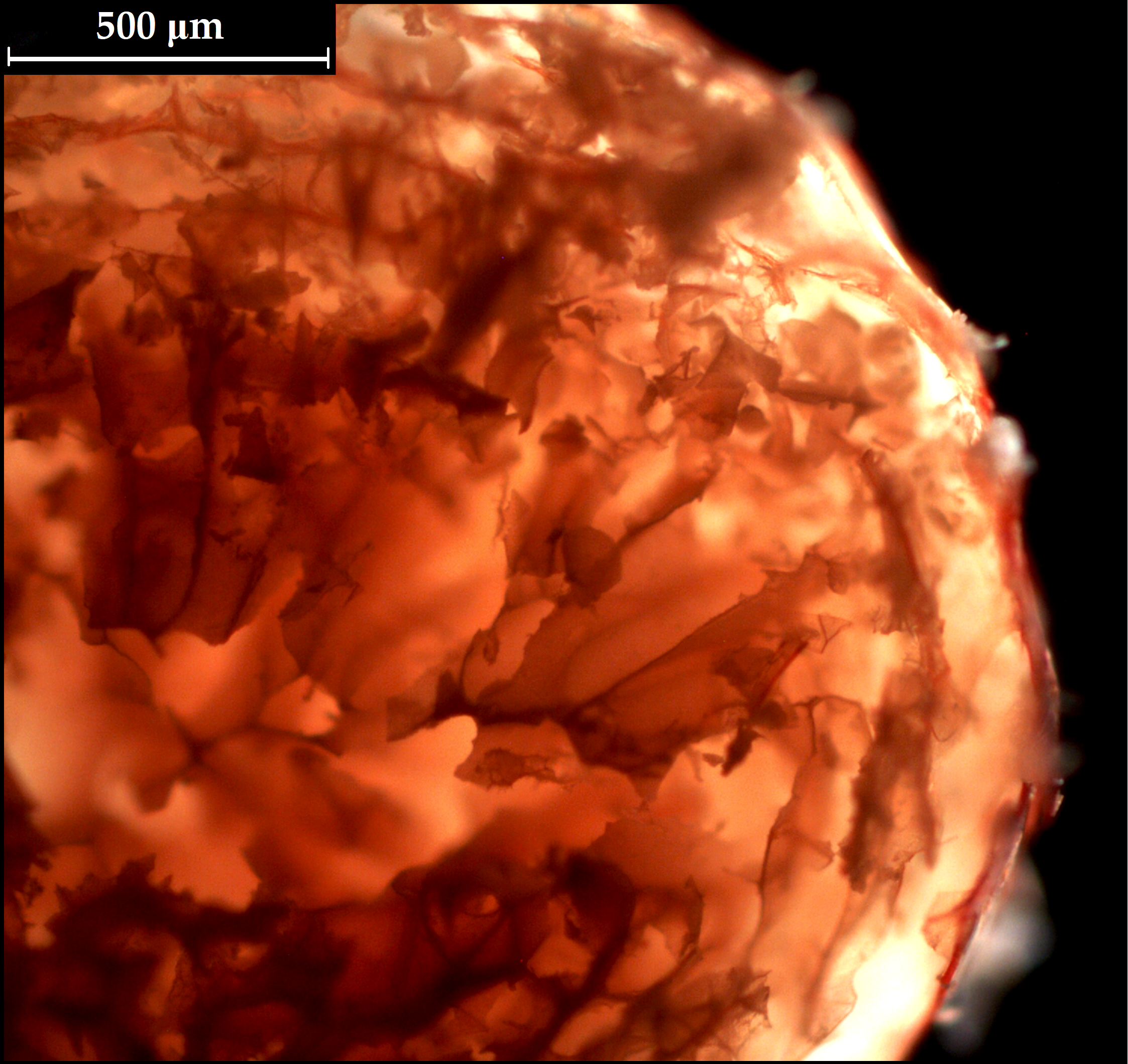 | 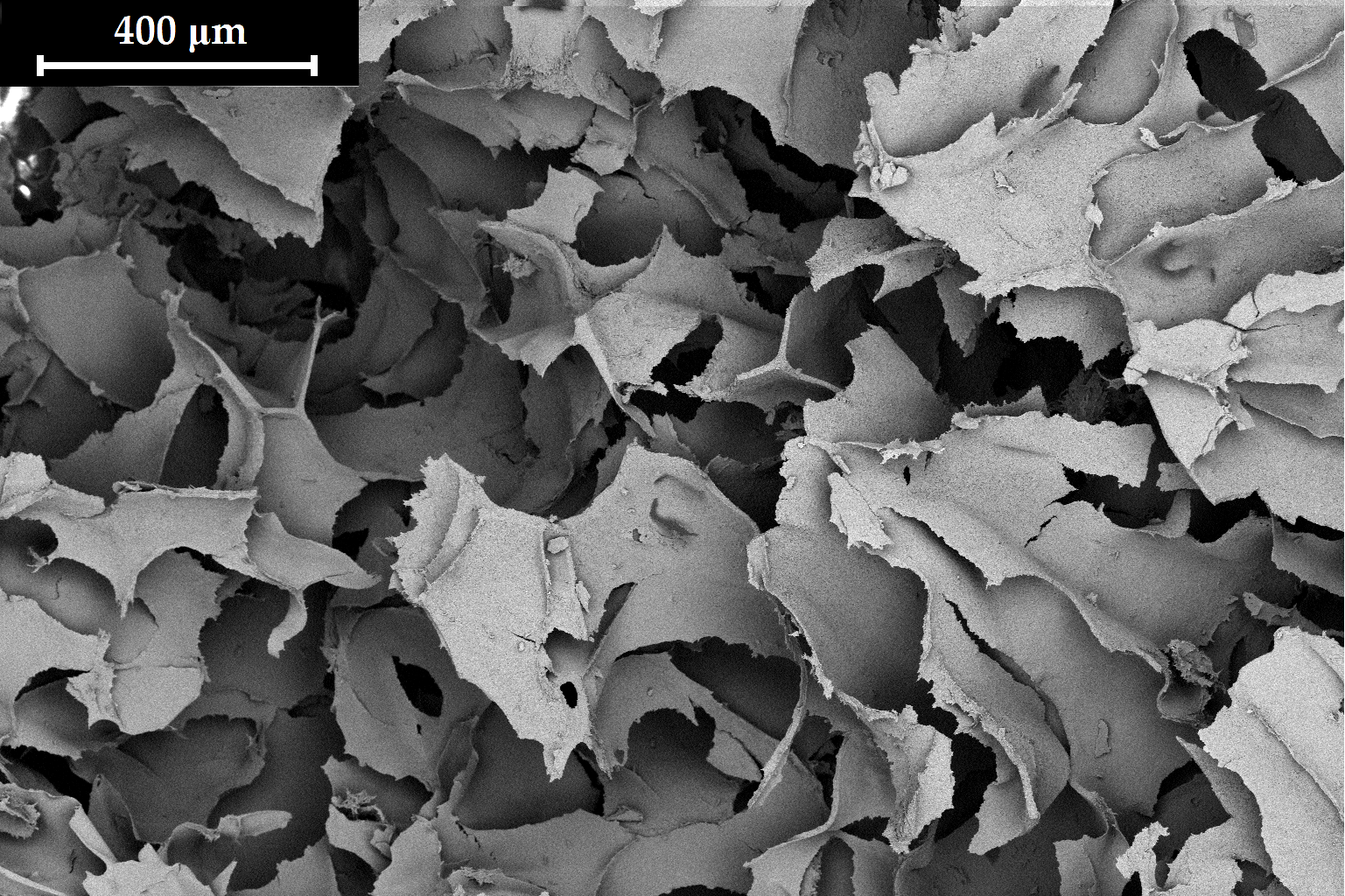 | 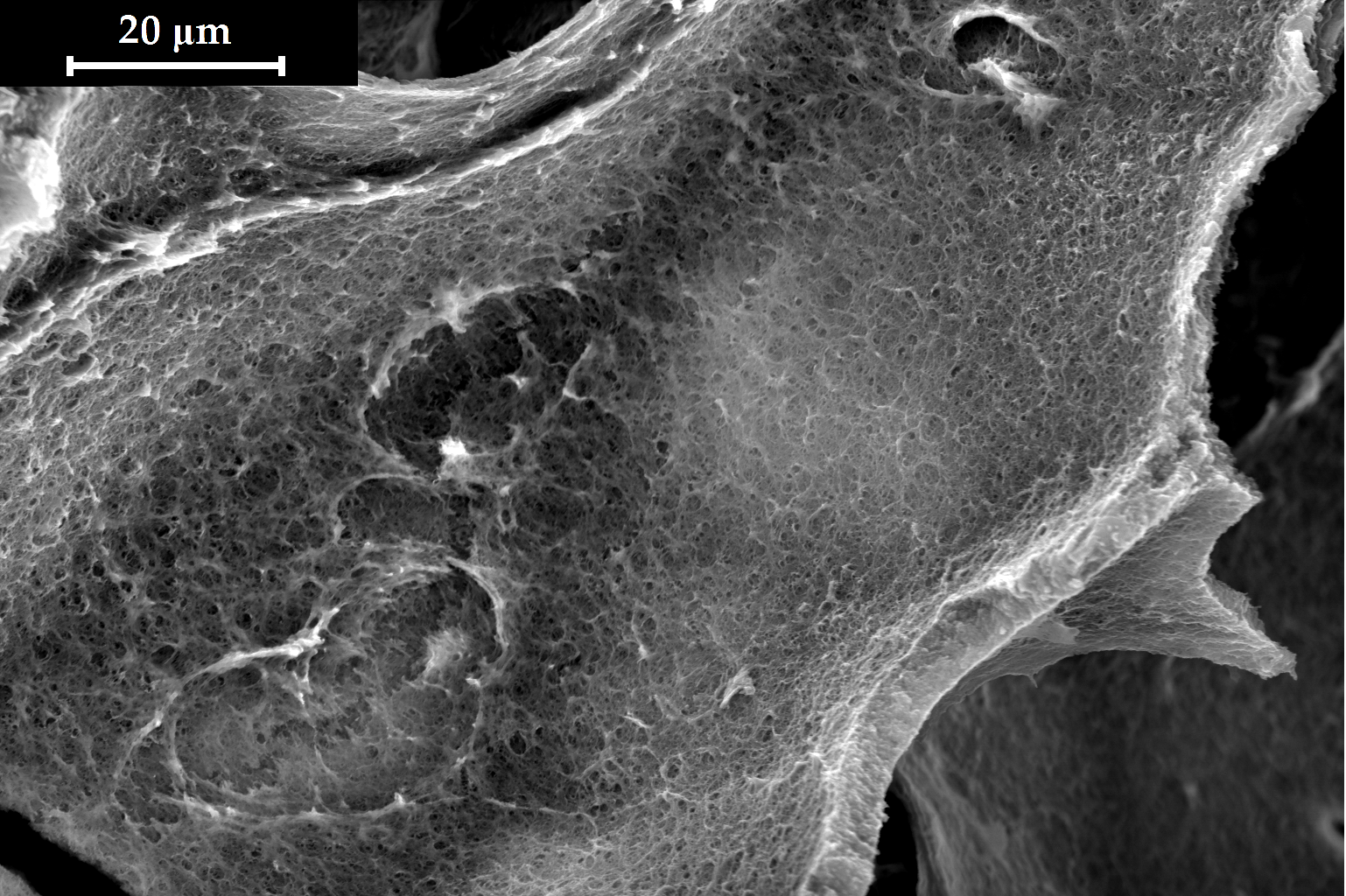 |
| --- | --- | --- |
| (**a**) | (**b**) | (**c**) |
| 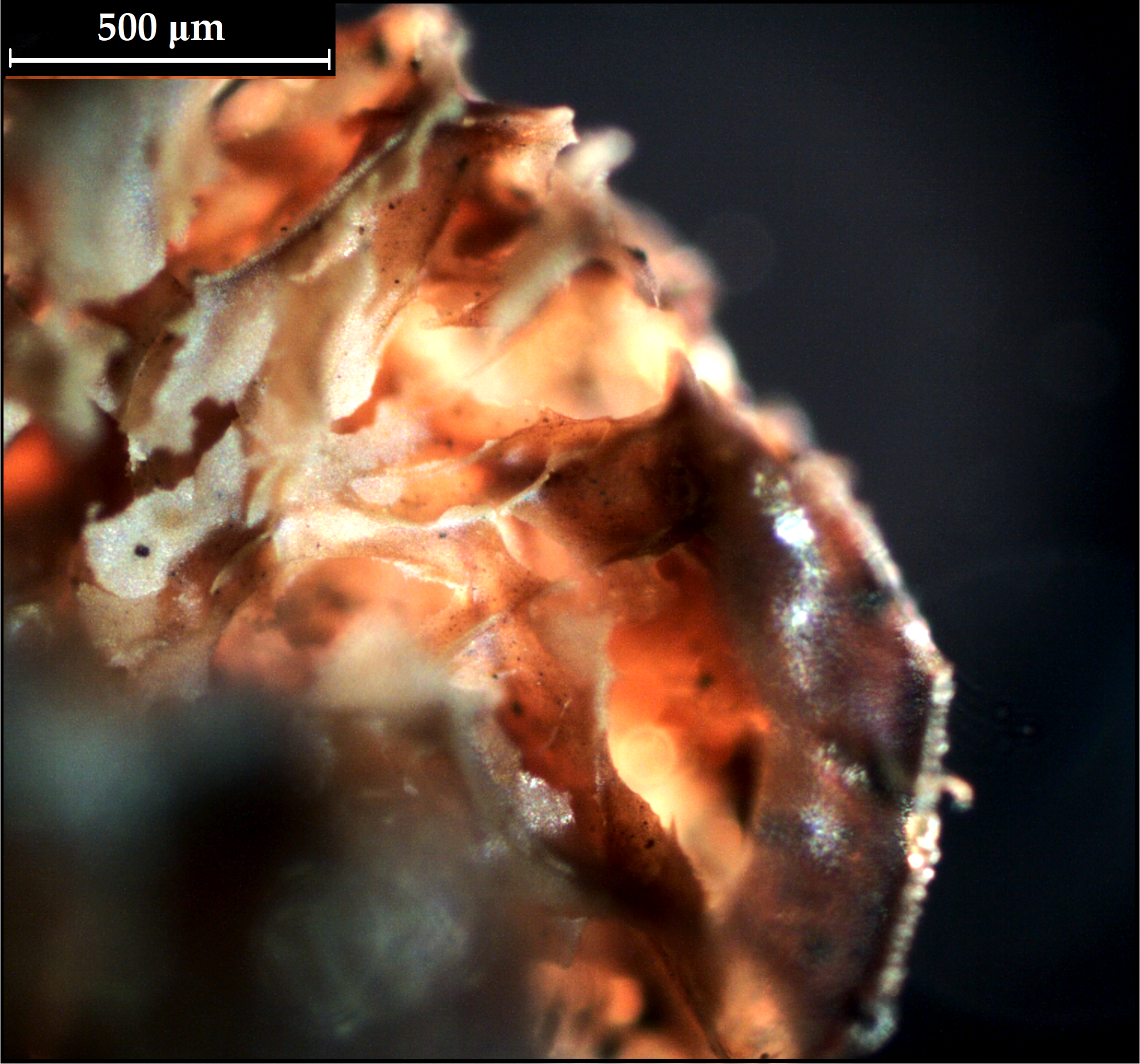 | 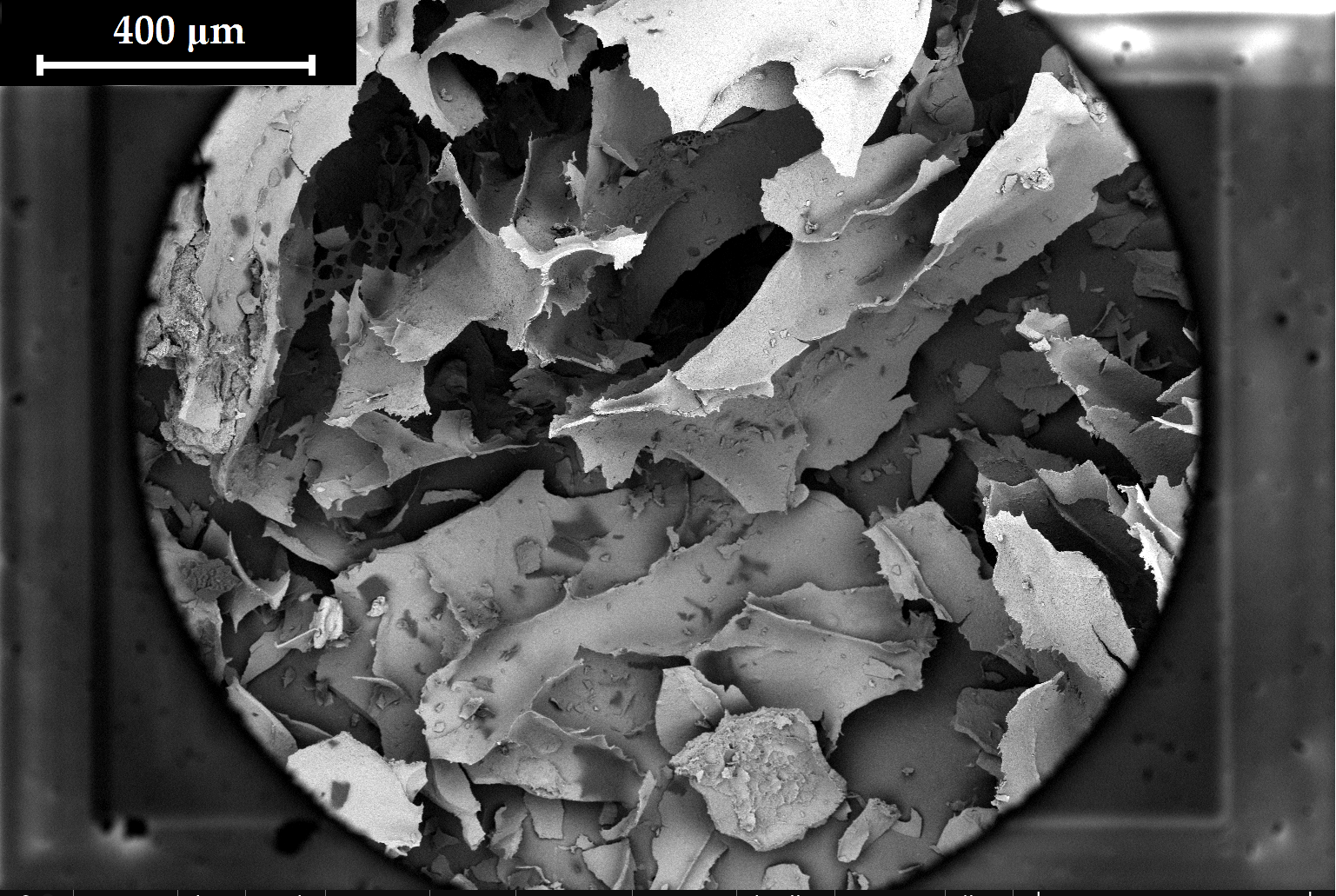 | 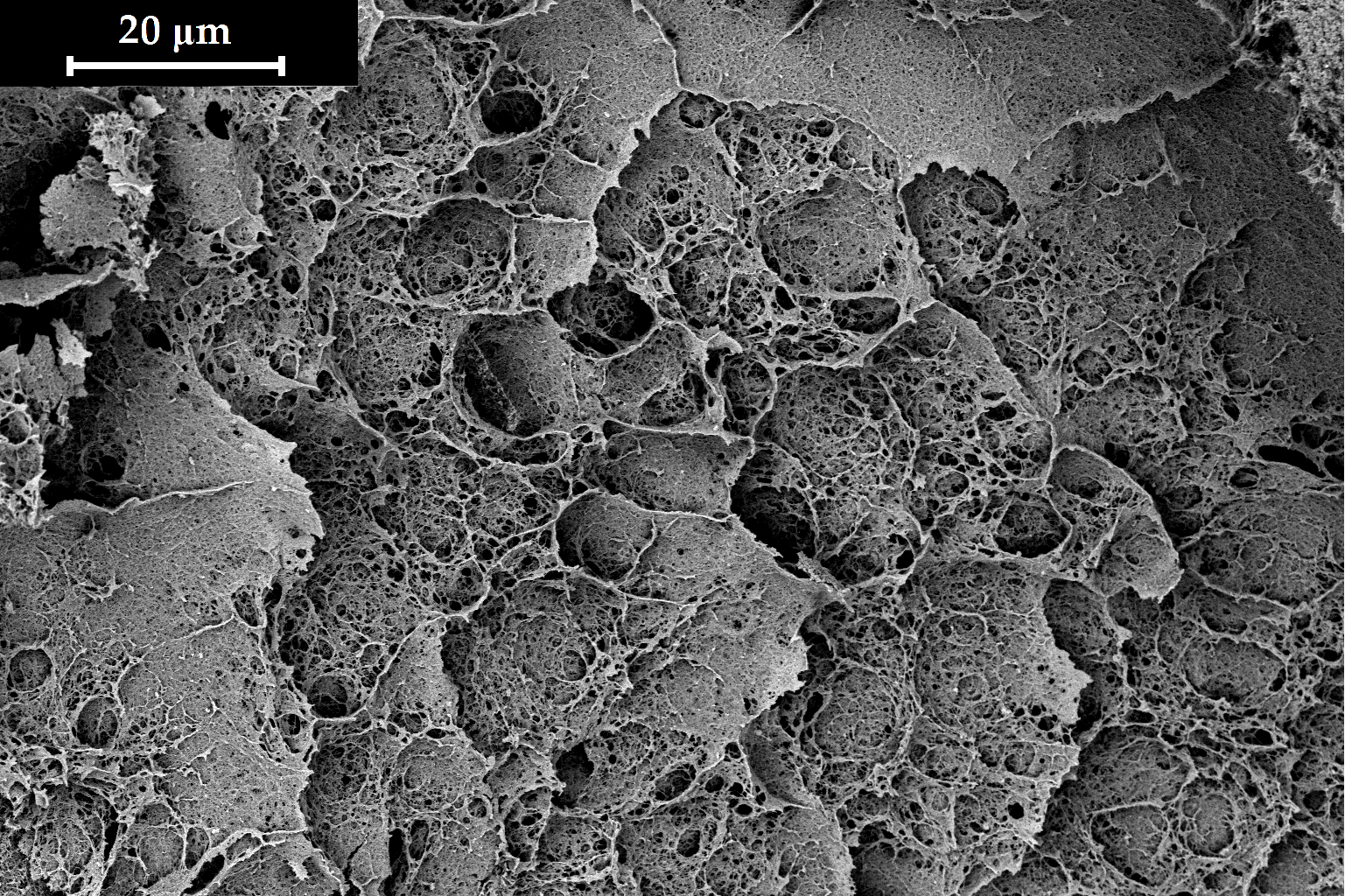 |
| (d) | (e) | (f) |
| 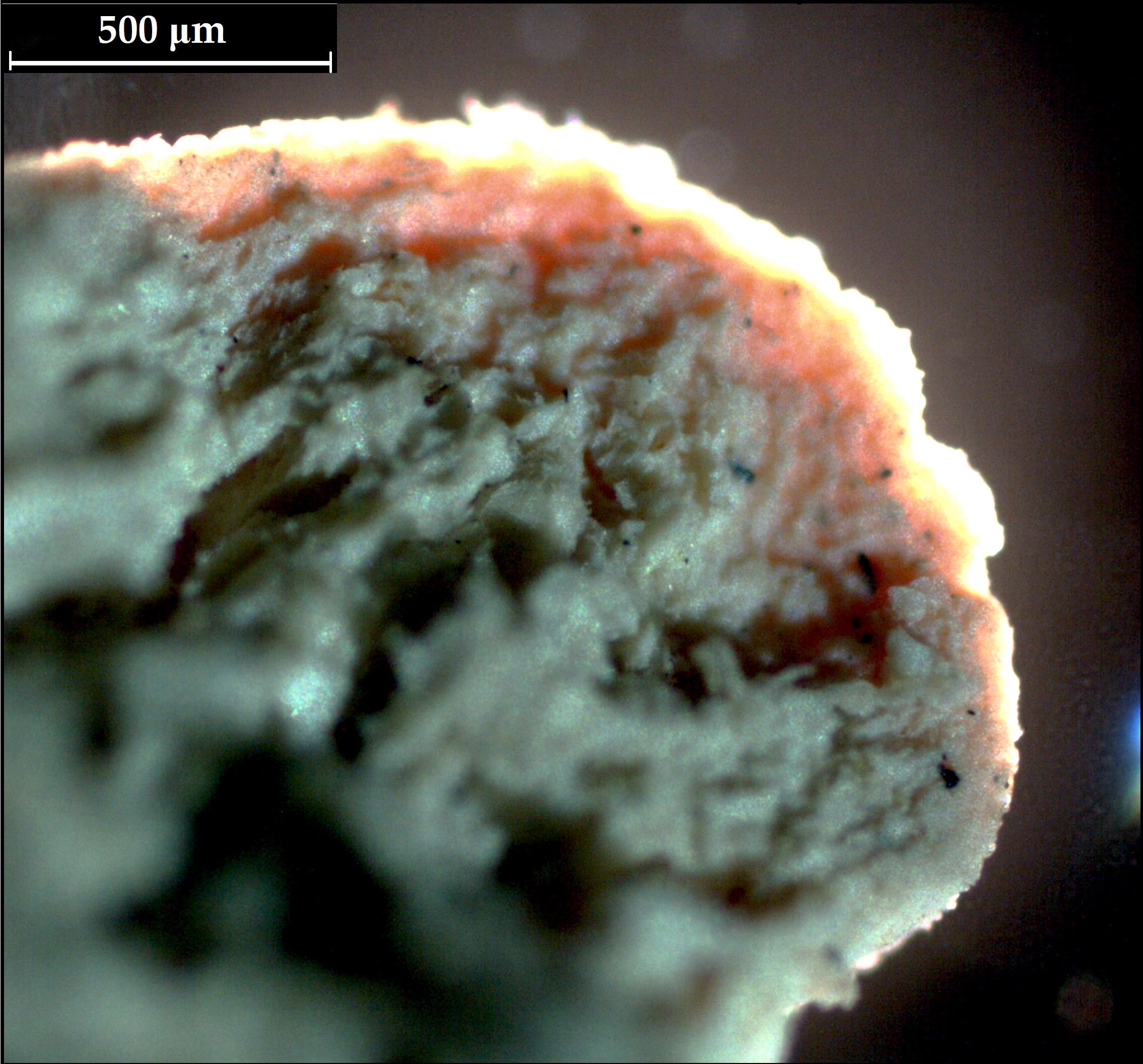 | 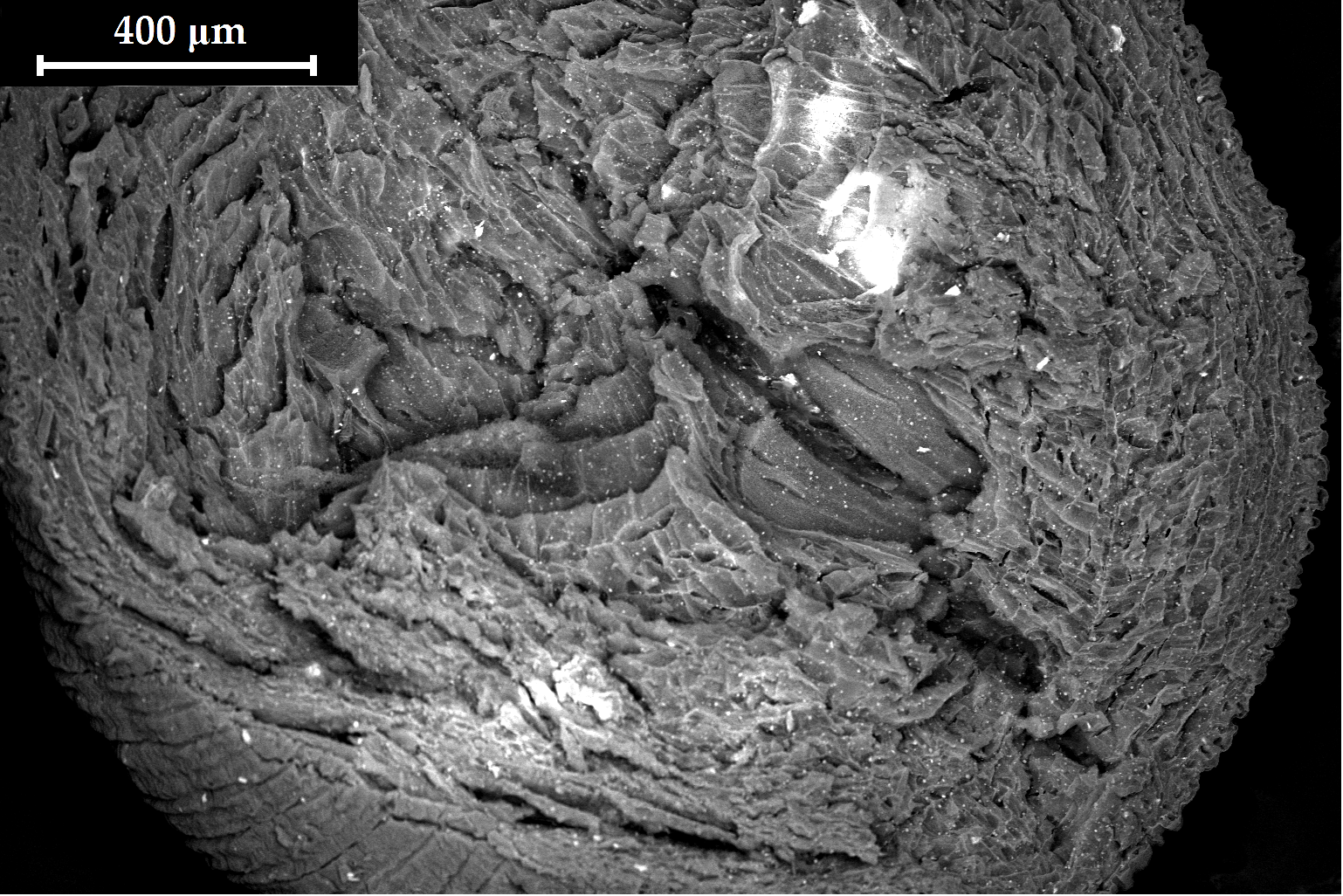 | 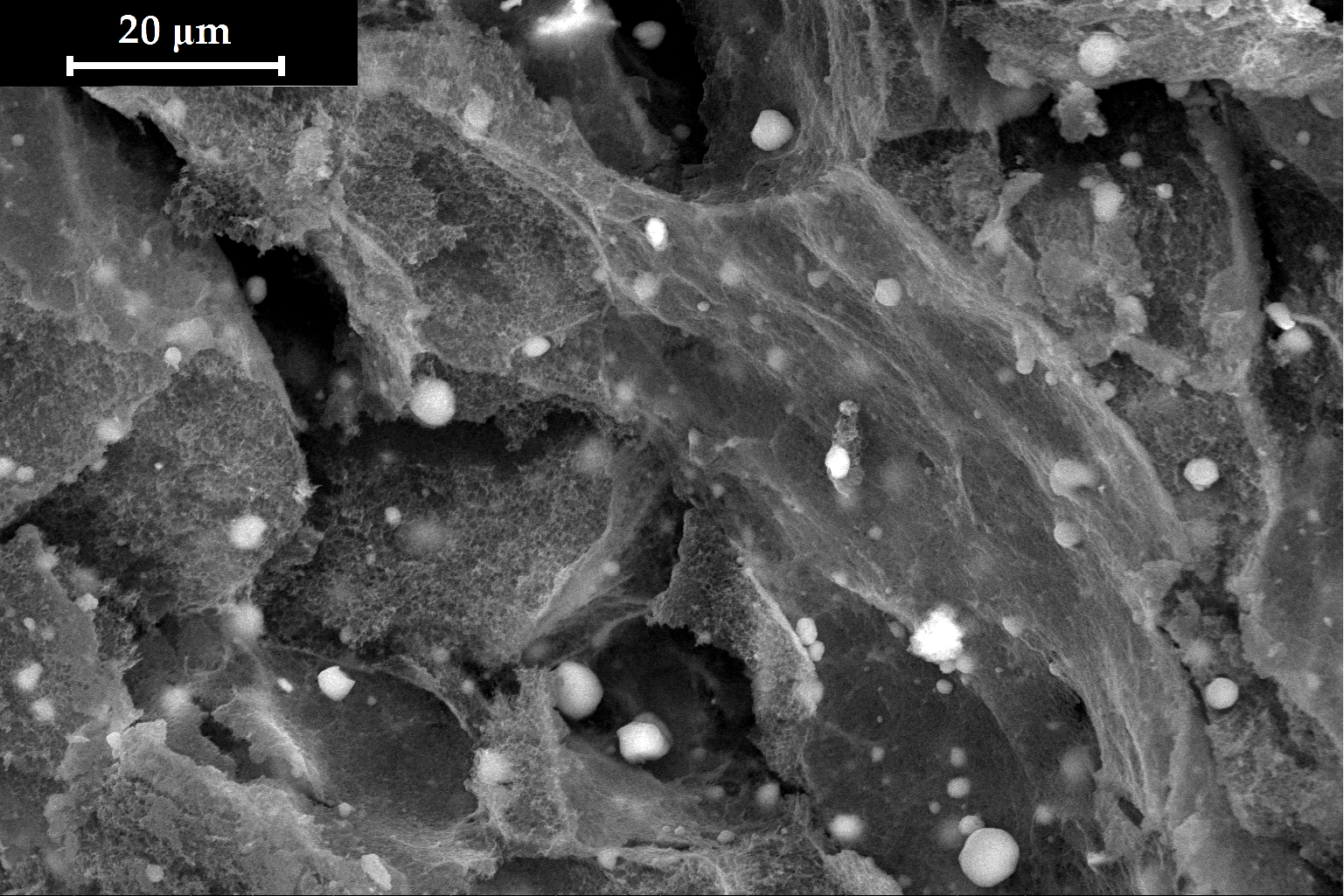 |
| (g) | (h) | (i) |
| 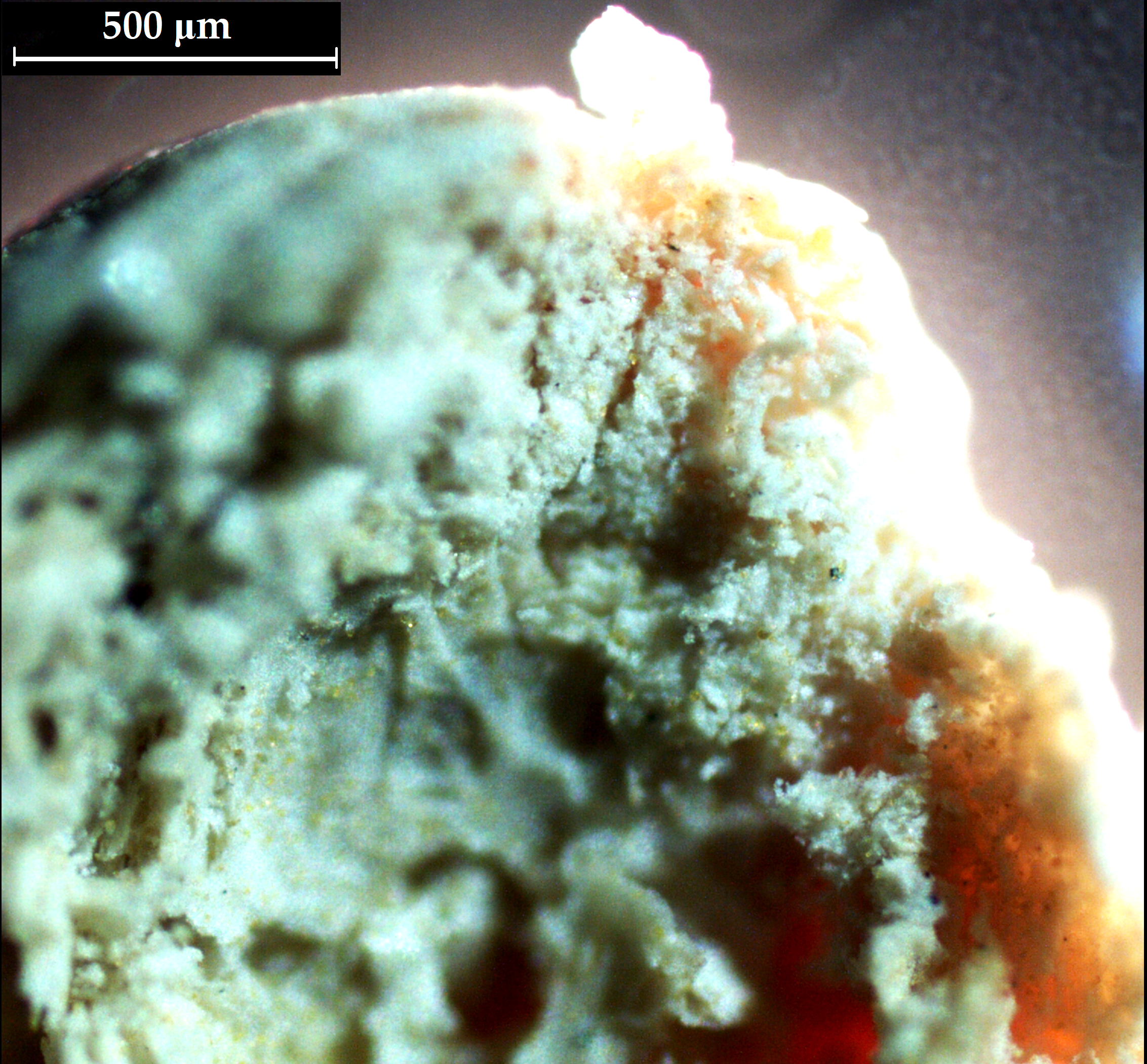 | 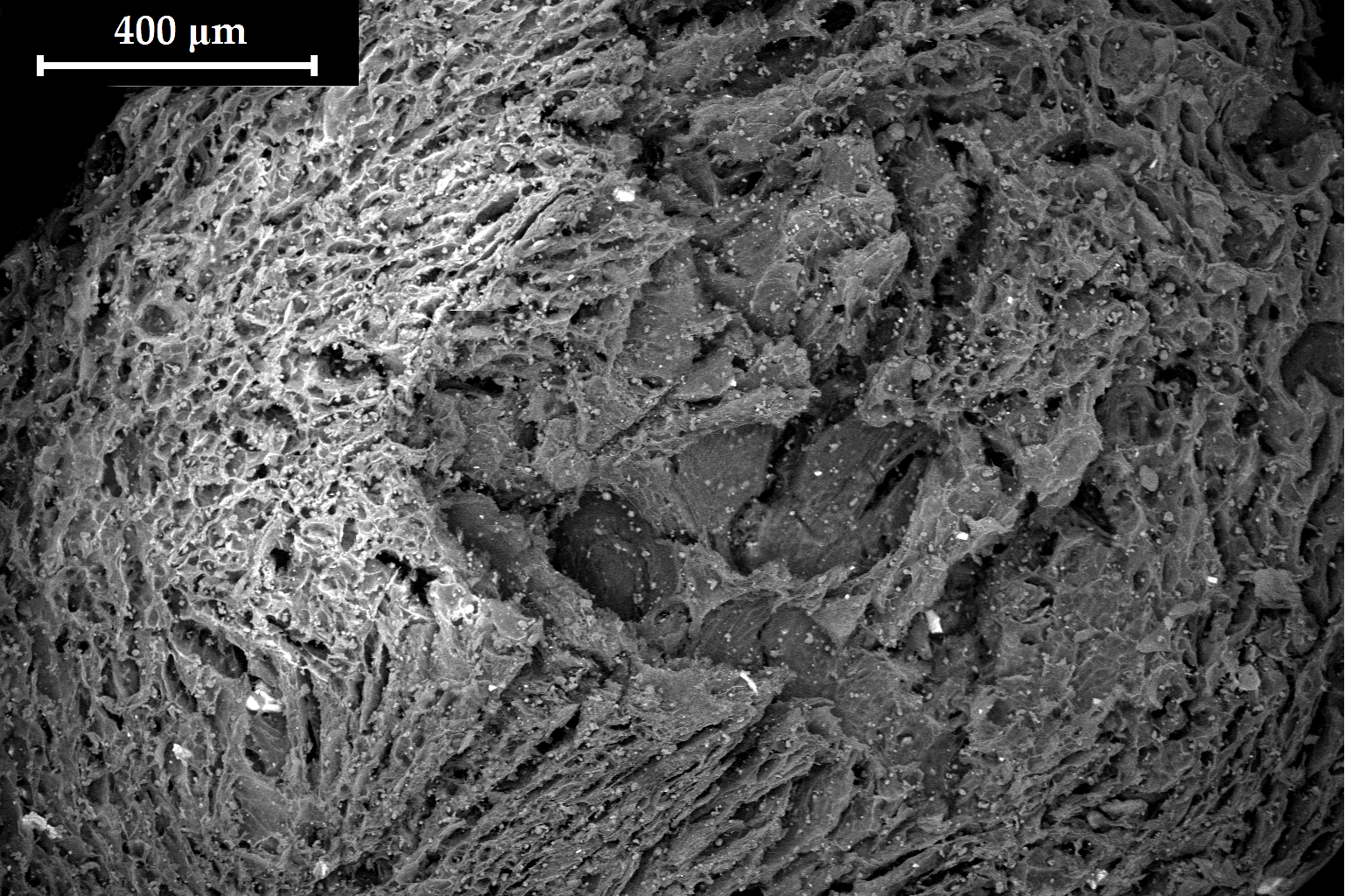 | 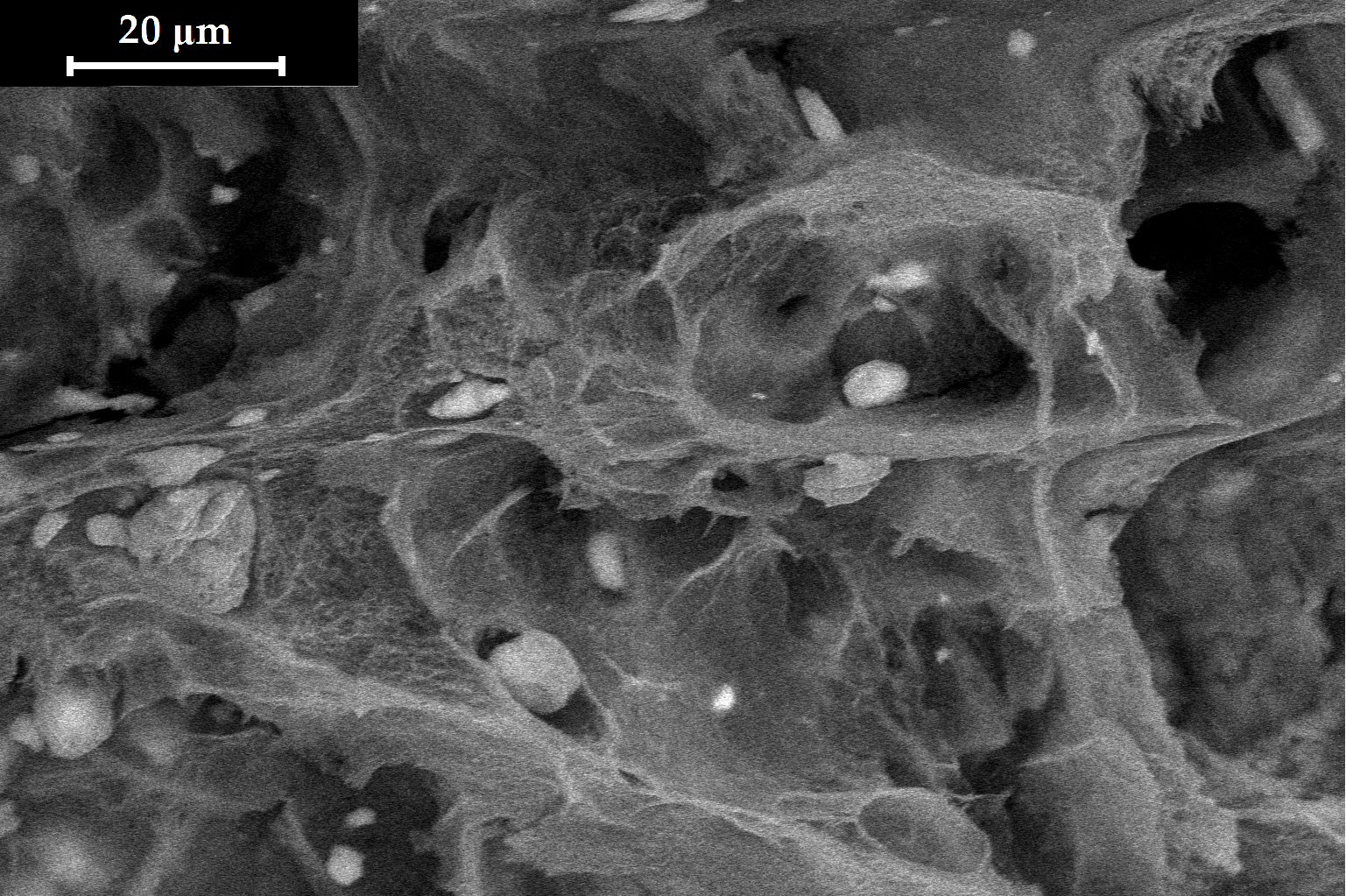 |
| (j) | (k) | (l) |
| 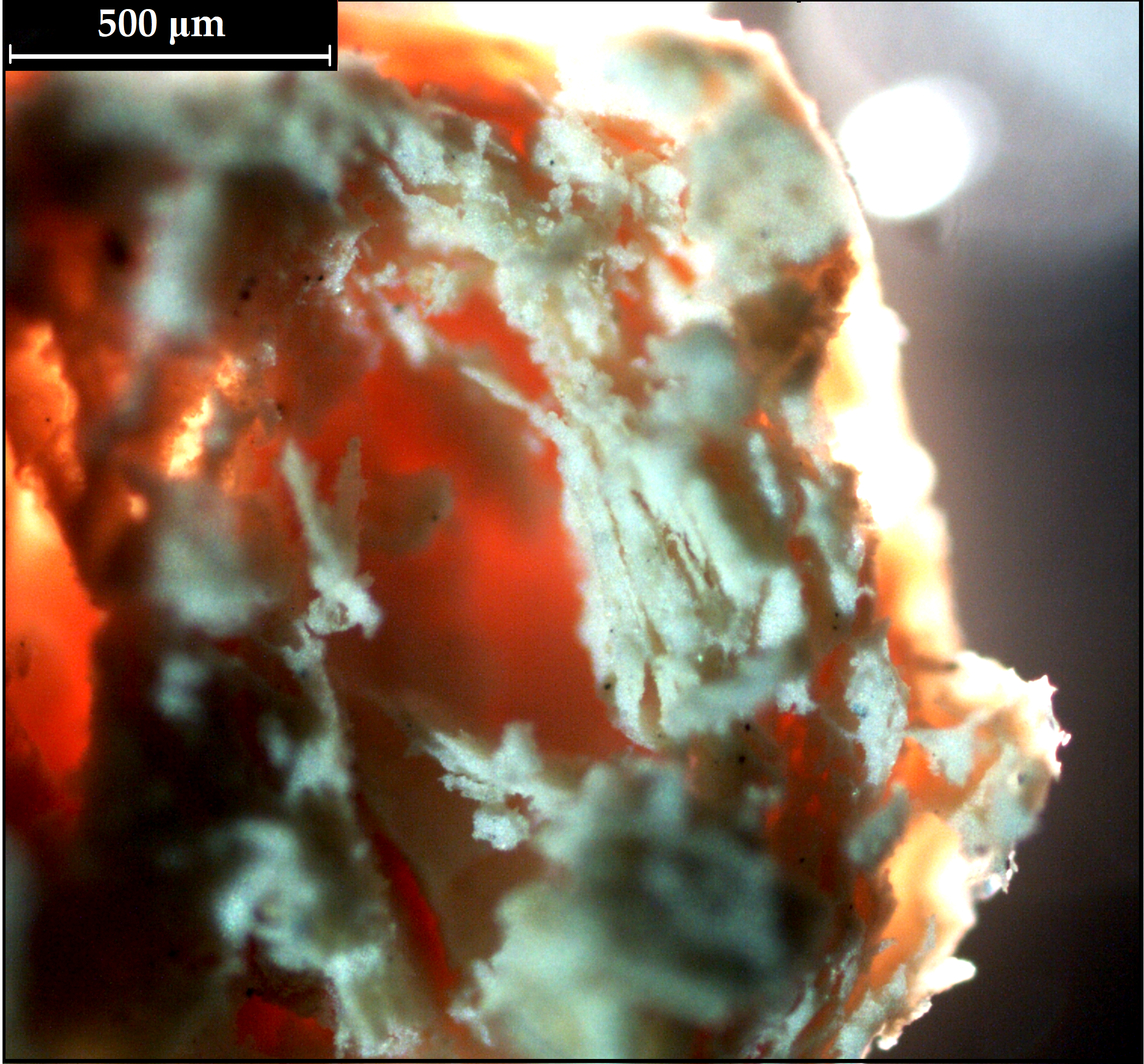 | 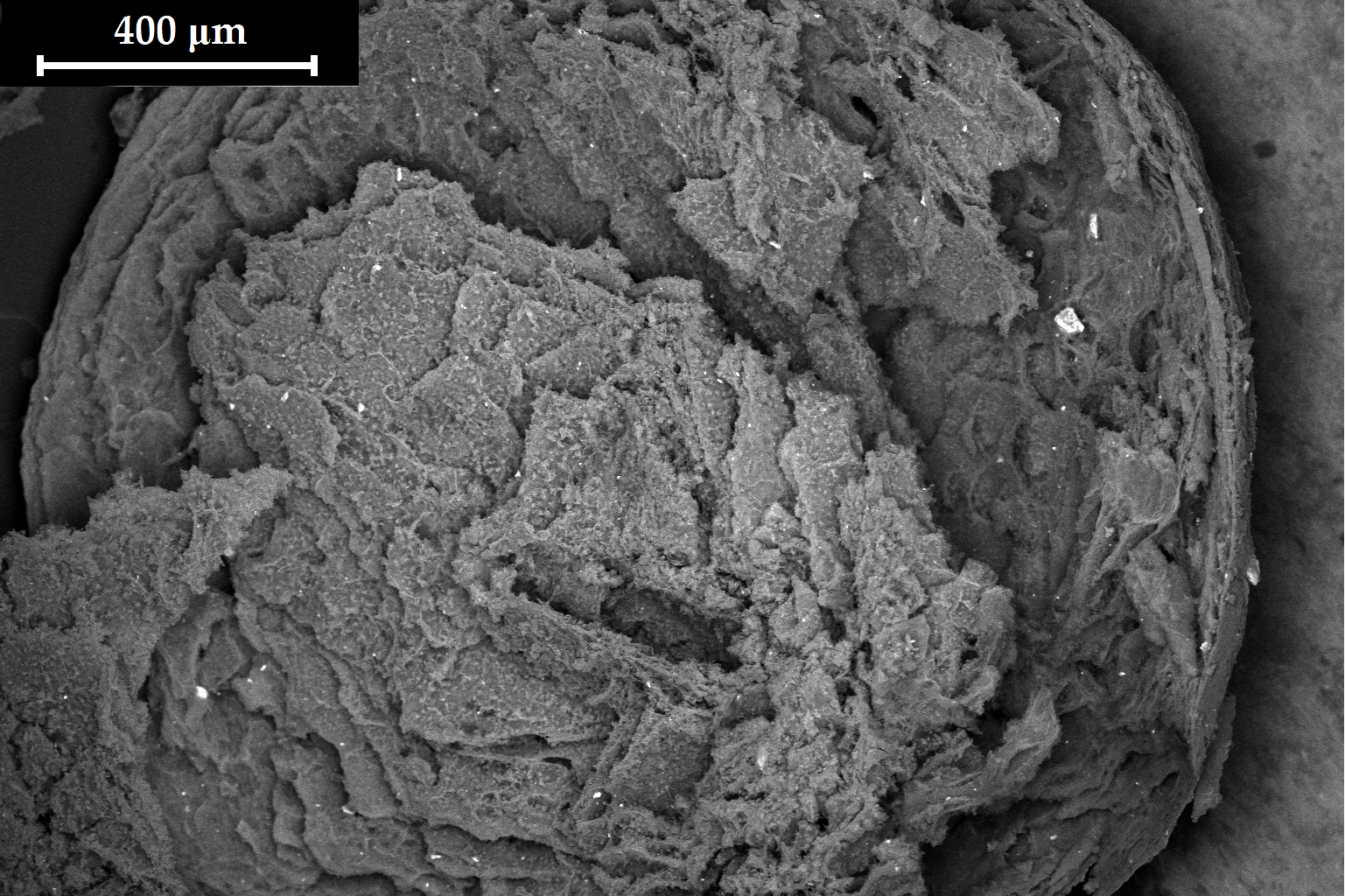 | 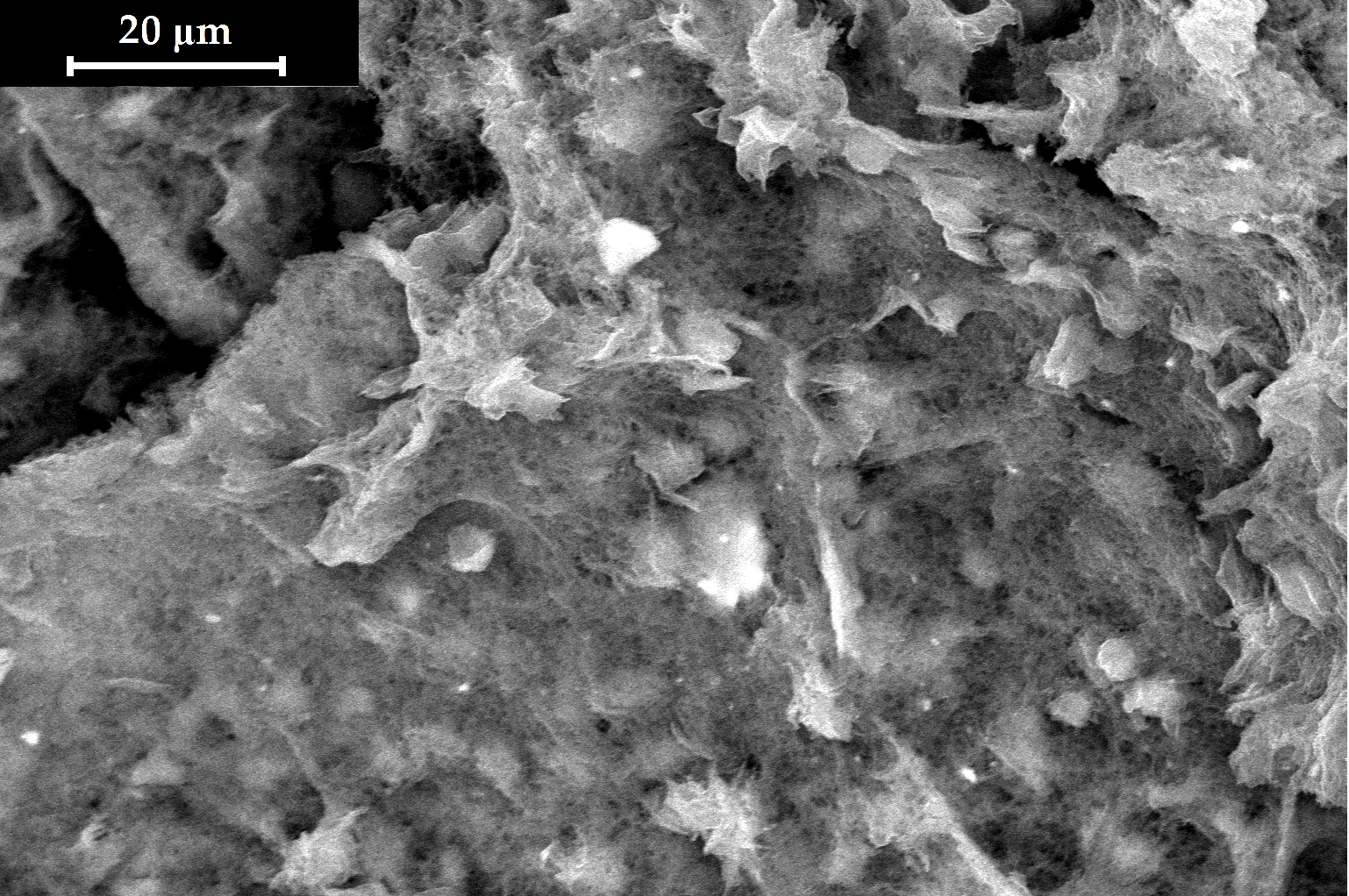 |
| (m) | (n) | (o) |

**Figure S2.** Cross-section morphology of CMC (**a**-**c**); CMC-Mn (**d**-**f**); CMC-Mn-S1 (**g**-**i**); CMC-Mn-S2 (**j**-**l**); CMC-Mn-S3 (**m**-**o**), obtained by polarized light microscopy and SEM, respectively.

| 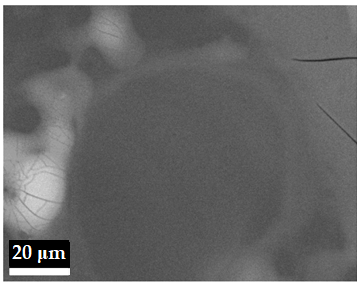 | 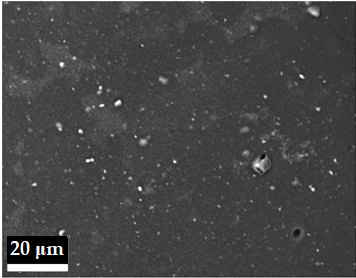 | 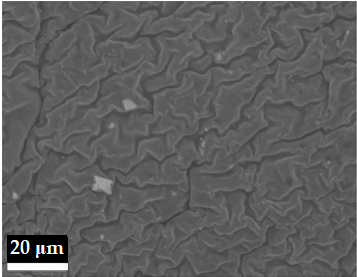 |
| --- | --- | --- |
| (**a**) | (**b**) | (**c**) |
| 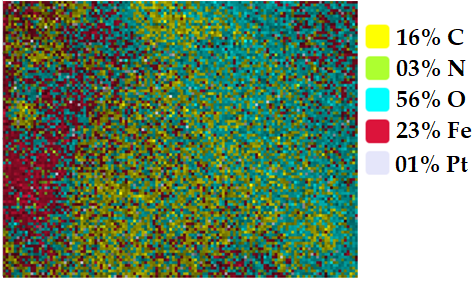 | 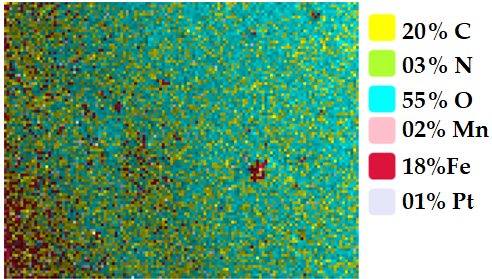 | 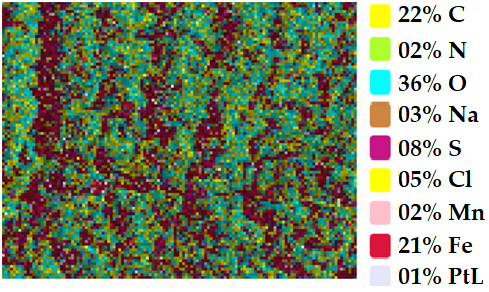 |
| (**d**) | (**e**) | (**f**) |
| 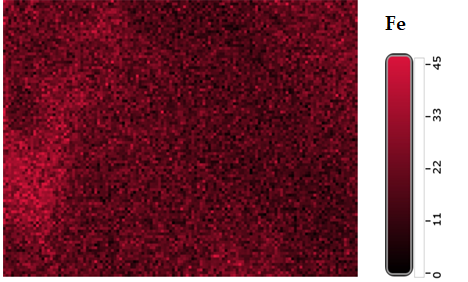 | 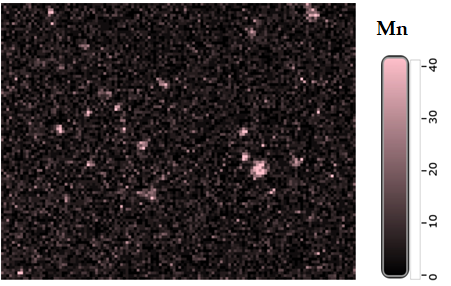 | 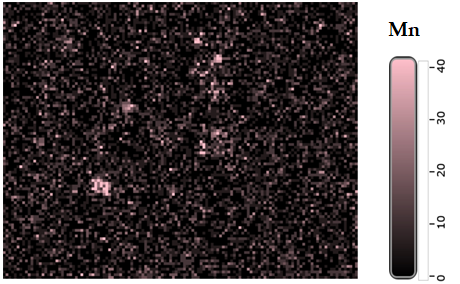 |
| (**g**) | (**h**) | (**i**) |

**Figure S3.** SEM images of CMC (**a**), CMC-Mn (**b**) and CMC-Mn-S1 (**c**) on the beads surface; corresponding elemental mapping and weight percentage of the constituent elements of the analyzed SEM surfaces (**d**-**f**), and the distribution of Fe (**g**), and Mn element in samples (**h**-**i**).

**Table S1.** Kinetic models, non-linear equations and parameters for MB dye adsorption onto CMC-Mn-S2 adsorbent, using different sorbent doses (*SD*); experimental conditions: T = 300 K, C_0_ = 50 mg/L.

|  | **Kinetic Model** | **Non-Linear Equation**^1^ | ***SD***  **(g/L)** | **Kinetic parameters**^2^ |  |  |  |  |
| --- | --- | --- | --- | --- | --- | --- | --- | --- |
|  | **PFO** | $q_{t}=q_{e}\left( 1-e^{-k_{1}t} \right)$ | *0.5* | *q_e_* = 63.12 (mg/g)  *k_1_* = 0.32 × 10^-1^  *χ^2^* = 1.56 |  |  |  |  |
|  |  |  | *1* | *q_e_* = 37.11 (mg/g)  *k_1_* = 0.58 × 10^-1^  *χ^2^* = 1.46 |  |  |  |  |
|  |  |  | *2* | *q_e_* = 19.42 (mg/g)  *k_1_* = 0.81 × 10^-1^  *χ^2^* = 0.40 |  |  |  |  |
|  |  |  | *3* | *q_e_* = 13.12 (mg/g)  *k_1_* = 0.12  *χ^2^* = 0.18 |  |  |  |  |
|  | **PSO** | $q_{t}=\frac{k_{2}q_{e}^{2}t}{1+k_{2}q_{e}t}$ | *0.5* | *q_e_* = 71.97 (mg/g)  *k_2_* = 0.52 × 10^-3^  *χ^2^* = 6.44 |  |  |  |  |
|  |  |  | *1* | *q_e_* = 40.25 (mg/g)  *k_2_* = 0.20 × 10^-2^  *χ^2^* = 3.28 |  |  |  |  |
|  |  |  | *2* | *q_e_* = 120.77(mg/g)  *k_2_* = 0.57 × 10^-2^  *χ^2^* = 0.60 |  |  |  |  |
|  |  |  | *3* | *q_e_* = 13.89 (mg/g)  *k_2_* = 0.13 × 10^-1^  *χ^2^* = 0.21 |  |  |  |  |

^1^  *q_e_* and *q_t_* (mg/g) represents the amounts of MB adsorbed on the CMC-Mn-S2 beads at the equilibrium and at time *t* (min), respectively; *k_1_* and *k_2_* are the rate constants of PFO and PSO models, respectively.

^2^ Chi-square statistic test (χ^2^) was determined by using equation S1:

| *χ^2^ = atiq_e_^(obs)^ - q_e_^(calc)^)^2^/ q_e_^(calc)^,* | (S1) |
| --- | --- |

where, *q_e_^(obs)^* denotes the observed (experimental) values and *q_e_^(calc)^* represents the calculated (theoretical) values of adsorption capacity (mg/g).

**Table S2.** Isotherm models, equations and parameters for MB dye adsorption onto CMC-Mn-S2 adsorbent (contact time: t = 300 min).

| **Temperature** |  | | **Isotherm Models and Equations** | |  |
| --- | --- | --- | --- | --- | --- |
|  | **Langmuir** ^1^ | **Freundlich** ^2^ | | **Dubinin-Raduschevich** ^3^ | |
|  | $q_{e}=\frac{q_{m}K_{L}C_{e}}{1+K_{L}C_{e}}$ | $q_{e}=K_{F}C_{e}^{1/n_{F}}$ | | $E_{S}=\frac{1}{\sqrt{{2K}_{D}}}$ | |
| **T = 300 K**  **(27 °C)** | *q_m_* = 247.91 (mg/g)  *K_L_* = 0.021 (L/mg)  *R_L_ =* 0.373  *χ^2^* = 286.50 | *K_F_* = 22.13  *n_F_* = 2.39  *χ^2^* = 62.98 | | *K_D_* = 4.20 × 10^−3^  *E_S_* = 10.91 (kJ mol^−1^)  *r^2^* = 0. 981 | |
| **T = 330 K**  **(57 °C)** | *q_m_* = 294.33 (mg/g)  *K_L_* = 0.006 (L/mg)  *R_L_ =* 0.614  *χ^2^* = 40.67 | *K_F_* = 6.75  *n_F_* = 1.68  *χ^2^* = 7.60 | | *K_D_* = 5.09 × 10^−3^  *E_S_* = 9.91 (kJ mol^−1^)  *r^2^* = 0. 984 | |

^1^ *q_e_* is the MB dye amount at the equilibrium (mg/g); *q_m_* is the theoretical maximum adsorption capacity (mg/g); *C_e_* is the equilibrium concentration of MB in solution (mg/L); *K_L_* is the Langmuir adsorption constant; R_L_ factor value represents the average of the calculated values for each initial dye concentrations (from 10 to 400 mg/L) [29], and *χ^2^* is calculated in accordance with equation S1.

^2^ *K_F_* represent the Freundlich constant and n_F_ is the surface heterogeneity factor.

^3^ *K_D_* is the Dubinin-Radushkevich parameter, *E_S_* represents the mean free energy of adsorption, and *r^2^* is the correlation coefficient


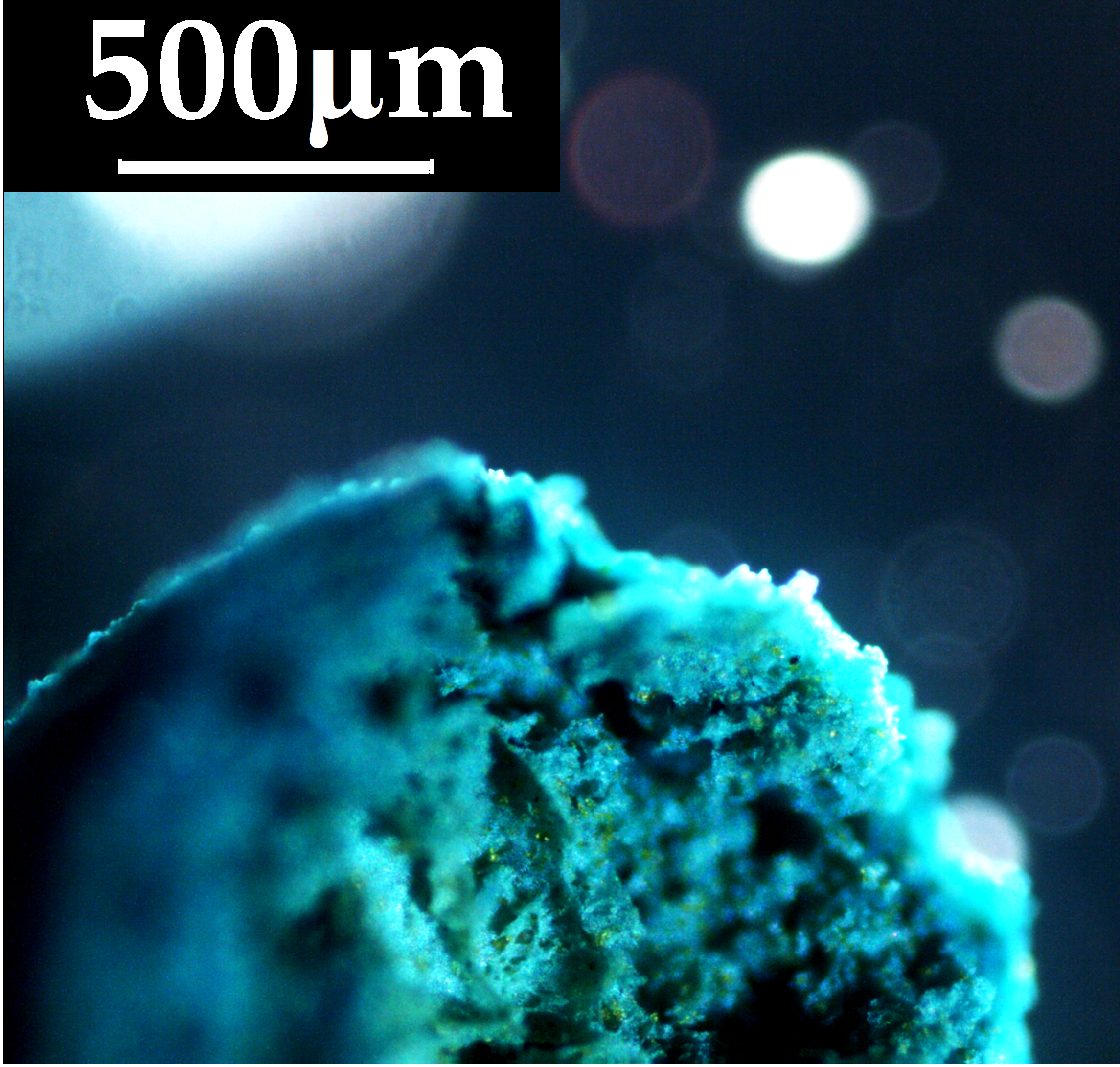


**Figure S4.** Image obtained by polarized light microscopy of CMC-Mn-S2 cross-section after MB adsorption.

**Table S3.** Comparison of the maximum adsorption capacities (q_e_^(obs)^)of CMC-based composite materials for retention of MB cationic dye.

| **CMC-Based Composites** | **Formulation** | **Maximum Adsorption Capacity (**q_e_^(obs)^, **mg/g)** | **Ref.** |
| --- | --- | --- | --- |
| CMC / polyvinyl alcohol / halloysite nanoclay | membrane | 8.29 mg/g | [54] |
| CMC /polyvinyl alcohol / turmeric | film | 6.27 mg/g | [55] |
| CMC / Fe_3_O_4_@SiO_2_ | nanoparticles | 29 mg/g | [26] |
| CMC / poly(acrylic acid-co-acrylamide) / Fe_3_O_4_ | particles | 34.3 mg/g | [56] |
| CMC / polyacrylic acid / graphene oxide | aerogel | 138 mg/g | [27] |
| CMC / ethylenediamine / graphene oxide | aerogel | 244.99 mg/g | [57] |
| CMC / carboxylated graphene oxide | microbeads | 180.32 mg/g | [25] |
| CMC / Al^3+^ / sodium n-dodecyl sulfate | beads | 75.46 mg/g | [29] |
| CMC / alginate / graphene oxide | beads | 78.5 mg/g | [58] |
| CMC / ascorbic acid / graphene oxide | beads | 222.72 mg/g | [59] |
| CMC-Mn-S2  (CMC / Manganese ferrite / sodium n-dodecyl sulfate / Fe^3+^)) | beads | 234 mg/g | This work |


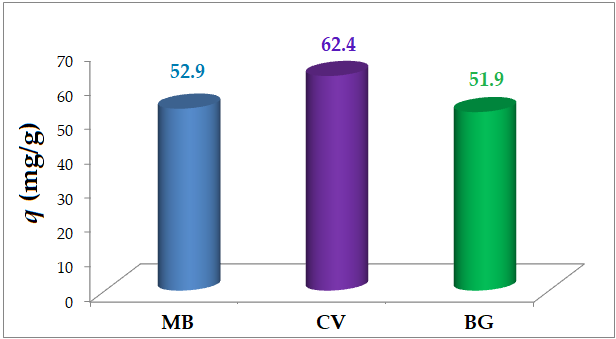


**Figure S5.** Adsorption capacities of Methylene Blue (MB), Crystal Violet (CV), and Brilliant Green (BG) onto CMC-Mn-S2 beads (experimental conditions: *SD* = 0.025, *C_0_* = 50 mg/g, *V* = 50 mL, *t* = 60 min).


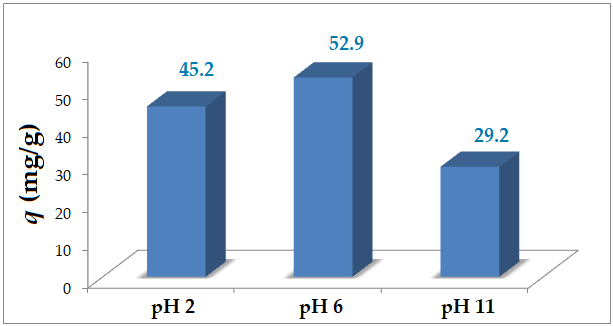


**Figure S6.** Adsorption capacities of Methylene Blue (MB) from aqueous solutions of pH 2 (adjusted with 0.1 M H_2_SO_4_) and pH 11 (adjusted with 0.1 M NaOH); experimental conditions: *SD* = 0.025, *C_0_* = 50 mg/g, *V* = 50 mL, *t* = 60 min.


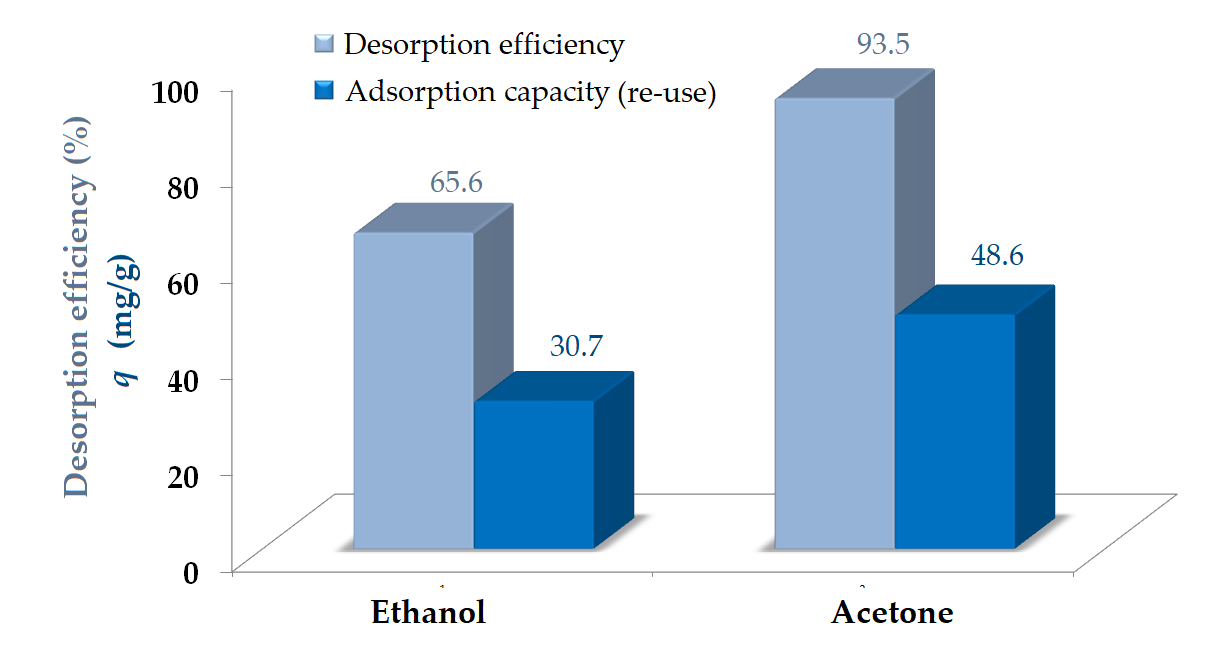


| (a) |
| --- |


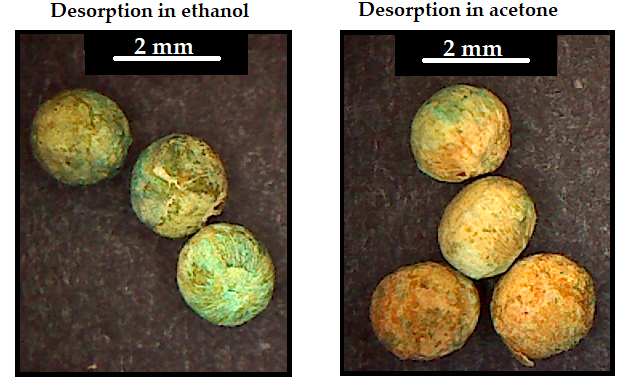


| (b) |
| --- |

**Figure S7.** (**a**) Desorption efficiency of MB in ethanol and acetone (experimental conditions: *SD* = 0.025, *V* = 50 mL, *t* = 24 h); and re-adsorption capacities (*q*, mg/g) of MB onto the recovered adsorbent (experimental conditions: *SD* = 0.025, *C_0_* = 50 mg/g, *V* = 50 mL, *t* = 60 min);)(**b**) macroscopic images of CMC-based beads after desorption in ethanol (left) and acetone (right).

Figure S7a shows the desorption efficiency of loaded CMC-Mn-S2 beads in two eluents (ethanol and acetone). It can be observed that the dye removal efficiency in acetone reached 93.5%, while in ethanol it was only 65.6%. This is also supported by the microscopic images given in Figure S7b, where it is possible to observe the MB being removed from the CMC sorbent surface in an effective manner. As expected, once a larger amount of MB was removed from the surface of the beads, a subsequent increased re-adsorption capacity of 48.6 mg/g was also recorded (in the case of the sorbent recovered from acetone).


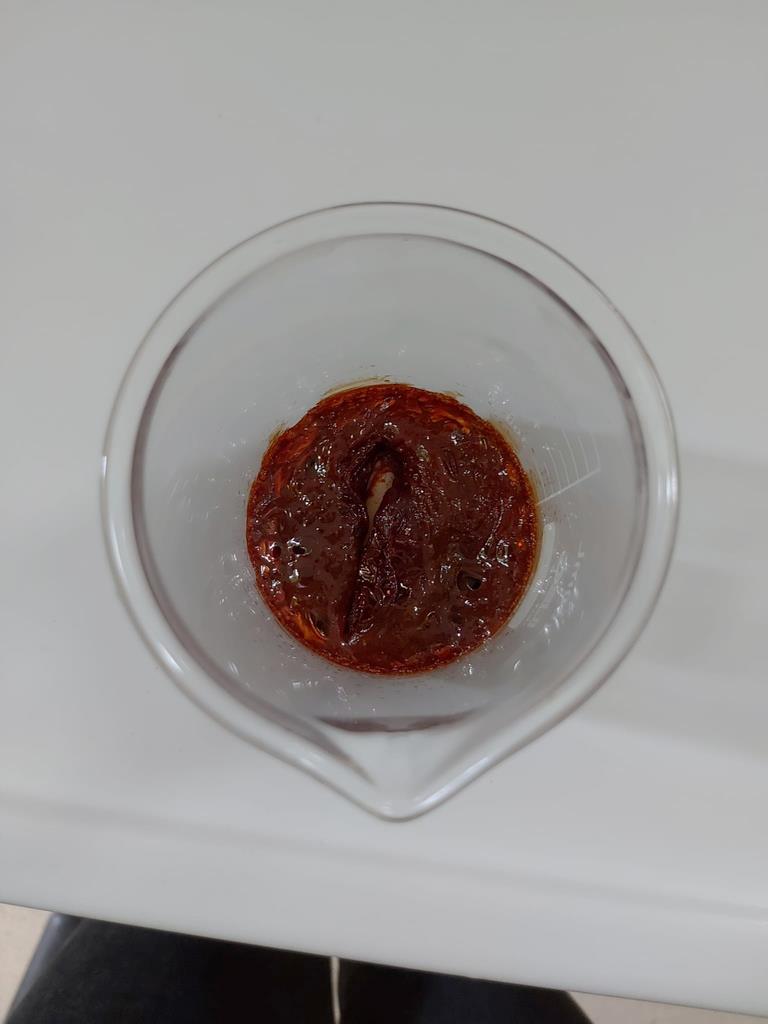

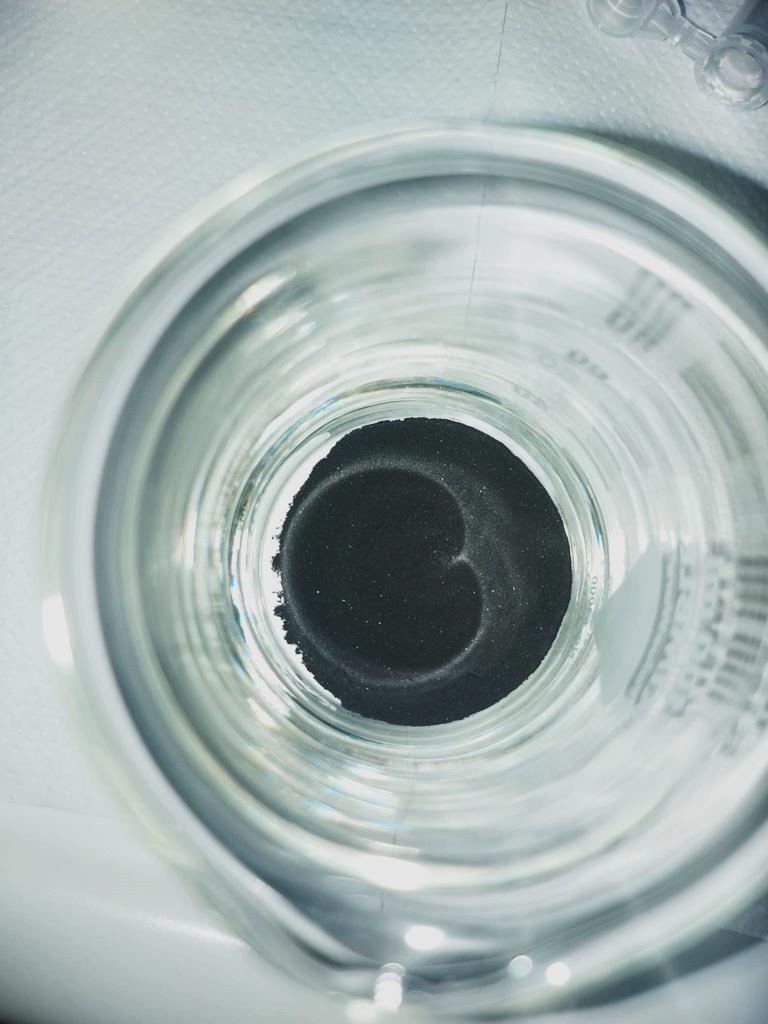


| (**a**) | (**b**) |
| --- | --- |

**Figure S8.** Representative images of the obtained (**a**) xerogel and (**b**) loose dark nanoparticles of manganese ferrite
